# Complete Chloroplast Genome Sequences of Mongolia Medicine *Artemisia frigida* and Phylogenetic Relationships with Other Plants

**DOI:** 10.1371/journal.pone.0057533

**Published:** 2013-02-27

**Authors:** Yue Liu, Naxin Huo, Lingli Dong, Yi Wang, Shuixian Zhang, Hugh A. Young, Xiaoxiao Feng, Yong Qiang Gu

**Affiliations:** 1 College of Life and Environmental Sciences, Minzu University of China, Beijing, China; 2 Institute of Chinese Materia Medica, China Academy of Traditional Chinese Medicine, Beijing, China; 3 Western Regional Research Center, Agricultural Research Service, United States Department of Agriculture (USDA-ARS), Albany, California, United States of America; 4 Department of Plant Science, University of California Davis, Davis, California, United States of America; 5 Graduate School, Minzu University of China, Beijing, China; J. Craig Venter Institute, United States of America

## Abstract

**Background:**

*Artemisia frigida* Willd. is an important Mongolian traditional medicinal plant with pharmacological functions of stanch and detumescence. However, there is little sequence and genomic information available for *Artemisia frigida*, which makes phylogenetic identification, evolutionary studies, and genetic improvement of its value very difficult. We report the complete chloroplast genome sequence of *Artemisia frigida* based on 454 pyrosequencing.

**Methodology/Principal Findings:**

The complete chloroplast genome of *Artemisia frigida* is 151,076 bp including a large single copy (LSC) region of 82,740 bp, a small single copy (SSC) region of 18,394 bp and a pair of inverted repeats (IRs) of 24,971 bp. The genome contains 114 unique genes and 18 duplicated genes. The chloroplast genome of *Artemisia frigida* contains a small 3.4 kb inversion within a large 23 kb inversion in the LSC region, a unique feature in Asteraceae. The gene order in the SSC region of *Artemisia frigida* is inverted compared with the other 6 Asteraceae species with the chloroplast genomes sequenced. This inversion is likely caused by an intramolecular recombination event only occurred in *Artemisia frigida*. The existence of rich SSR loci in the *Artemisia frigida* chloroplast genome provides a rare opportunity to study population genetics of this Mongolian medicinal plant. Phylogenetic analysis demonstrates a sister relationship between *Artemisia frigida* and four other species in Asteraceae, including *Ageratina adenophora, Helianthus annuus, Guizotia abyssinica* and *Lactuca sativa,* based on 61 protein-coding sequences. Furthermore, *Artemisia frigida* was placed in the tribe Anthemideae in the subfamily Asteroideae (Asteraceae) based on *ndhF* and *trnL-F* sequence comparisons.

**Conclusion:**

The chloroplast genome sequence of *Artemisia frigida* was assembled and analyzed in this study, representing the first plastid genome sequenced in the Anthemideae tribe. This complete chloroplast genome sequence will be useful for molecular ecology and molecular phylogeny studies within *Artemisia* species and also within the Asteraceae family.

## Introduction

*Artemisia frigida* Willd., named as “Agi” in the Mongolian language, is an important Mongolian traditional medicinal plant [1], distributed widely in the Inner Mongolia Autonomous Region and the northern part of China. This plant has medicinal application for stanch and detumescence, so it is often used to care for bleeding, arthroncus, rheumatism, menoxenia, and other ailments [1]. Besides its medicinal efficacy, it is also valued as an important food resource for livestock, and a remarkable component of the desert ecosystem [1].

*Artemisia frigida* belongs to the largest genus in the tribe Anthemideae of the family Asteraceae, which is the second largest family of plants in the world, consisting of over 20,000 species [2]. *Artemisia frigida* is a diploid species (2n = 2X = 18) and its haploid genome size is estimated to be 2,567 Mb [3]. However, polyploid *A. frigida* species with 2n = 4X = 36 have been identified in nature [4]. In recent years, there has been extensive research focused on the medicinal and pharmacological aspects and effects of the *Artemisia frigida* plant [5]–[9]. However, there has not been a comprehensive study of the genetic variability found in natural populations [1]. With the increasing demand for commercial use and the important ecological value of this traditional medicinal plant, large-scale breeding efforts need to be developed for *Artemisia frigida.* Selection of germplasm with high pharmaceutical efficacy at the molecular level is important and requires the availability of efficient genetic and molecular marker data. Access to genetic information will not only improve the genetic breeding process, but also will aid in downstream analysis of sequence data and improvement of *Artemisia frigida*’s medicinal qualities. Currently, there are only 24 sequences available for *Artemisia frigida*, including 6 nrDNA sequences and 18 chloroplast DNA sequences listed in GenBank [10]–[16] (http://www.ncbi.nlm.nih.gov/nuccore/?term=Artemisia%20frigida%20). Therefore, there is a clear need to develop genomic resources for *Artemisia frigida* in order to efficiently apply molecular and biotechnological approaches for the improvement of its value as an important medicinal plant.

Chloroplasts are plant organelles that contain the entire enzymatic machinery necessary for photosynthesis and other biochemical pathways. Most land plants have a highly conserved chloroplast genome organized into a single circular chromosome [17] that contains two copies of an inverted repeat (IR) separating a large single copy region (LSC) and a small single copy region (SSC). To date, over 200 chloroplast (cp) genome sequences are available in The Chloroplast Genome Database (http://chloroplast.ocean.washington.edu/cpbase/run). The vast majority of angiosperm cp genomes are highly conserved [18]. However, the gene order found in the LSC region of the Asteraceae, Fabaceae, and Poaceae families [19]–[21] is reversed when compared with *Nicotiana tabacum* [22], due to the presence of a large inversion in the Asteraceae, Fabaceae, and Poaceae family [19]–[21]. These structural differences in cp genomes can be exploited in the phylogenetic classification and molecular improvement of plants like *Artemisia frigida*. In addition, comparative analysis of cp genomes from distant and closely related species will not only allow for understanding the molecular evolution of cp genomes, but also facilitate the association of important traits controlled by plastid genomes.

One strategy for improving a plant species is through chloroplast genetic engineering to add high-value agronomic traits via transgenic expression [23], or to engineer multi-gene expression components in a single transformation event [23]–[25]. Plastid transformation, achieved via homologous recombination, is very advantageous compared to nuclear genome transformation mainly because it can generate high levels of expression and the recombinant DNA is more easily contained since chloroplasts are maternally inherited in most species of angiosperm [26]. Furthermore, chloroplast genetic engineering has also been widely used in basic research to understand plastid biogenesis and function [27]–[29].

Traditionally, sequencing of plastid genomes is done by isolation of chloroplasts followed by purification and amplification of plastid DNA for library construction and sequencing. Recently, a number of cp genome sequences are being reported using next-generation sequencing techniques due to the advantages of high-throughput, time-savings, and low-cost [30]–[33]. We report the complete cp genome sequence of *Artemisia frigida*, a kind of Mongolian traditional medicinal plant, using 454 pyrosequencing methods (Roche GS FLX+). We also describe details in the cp genome assembly, annotation, and comparative analysis with the sequences of cp genomes from other angiosperm species, including the six completed Asteraceae cp genomes. we identified and characterized a unique sequence rearrangement event in the *Artemisia frigida* cp genome, which resulted in the inversion of gene order in the SSC region as compared with other Asteraceae species. This work will lay a foundation for the molecular biology study and genetic improvement of *Artemisia frigida* in the future.

## Methods

### DNA Sequencing

A wild diploid *Artemisia frigida* (accession number NM1) from our germplasm collection from the Naimanqi area in Inner Mongolia Autonomous Region, China, was used for total DNA isolation from one gram of leave tissue using the DNeasy Plant Mini Kit (Qiagen, CA, USA). The DNA (1 µg) was sheared by nebulization, subjected to 454 library preparation and shotgun sequencing using the Genome Sequencer (GS) FLX+ platform [34] at the in-house facility (USDA-ARS, Western Regional Research Center, USA). The obtained nucleotide sequence reads were assembled using the GS *De Novo* Assembler version 2.6 and visualized by CONSED [35]. The assembled sequences and unassembled sequences were analyzed by BlastN and BlastX program against GenBank cp genome data to find *Artemisia frigida* cp genome sequence.

### Genome Analysis

The genome was annotated using the program DOGMA (Dual Organellar GenoMe Annotator [36]). The predicted annotations were verified using BLAST similarity search [37]. All genes, rRNAs and tRNAs were identified using the plastid/bacterial genetic code. The frequency of codon usage was calculated from exon sequences of all protein-coding genes in the *Artemisia frigida* genome. Inversions in the *Artemisia frigida* cp genome were identified by comparison to the sequence in the inverted region of *Lactuca sativa* (DQ383816) [38], *Helianthus annuus* (NC_007977) [38], and *Nicotiana tabacum* (NC_001879) [22]. Comparison of *Artemisia frigida* cp genome structures with *Lactuca sativa*, *Helianthus annuus, Guizotia abyssinica* (NC_010601) [39], *Parthenium argentatum* (NC_013553) [40], *Ageratina adenophora* (NC_015621) [41], and *Jacobaea vulgaris* (NC_015543) [42], which are all in the Asteraceae family, was performed using the mVISTA program in Shuffle-LAGAN mode [43], using the sequence annotation information of *Artemisia frigida*.

The gene orders in SSC regions of *Artemisia frigida* were compared with the above 6 Asteraceae species and 4 other species including *Nicotiana tabacum* (Solanaceae) (eudicots), *Piper cenocladum* (magnoliids) (NC_008457) [44], *Dioscorea elephantipes* (Dioscoreaceae) (monocots) (NC_009601) [45], and *Chloranthus spicatus* (Chloranthaceae) (NC_009598) [45].

REPuter [46] was used to identify and locate forward, palindrome, reverse, and complement sequences with n ≥30 bp and a sequence identity ≥90%. We ran the same REPuter analyses against the other 6 Asteraece species chloroplast genomes that were used for mVISTA to assess the relative number of repeats in chloroplast genomes. Microsatellite markers were predicted using MISA [47]. In the search for SSR standards, we defined SSRs as mononucleotide repeats ≥10 bases, dinucleotide repeats ≥12 bases, trinucleotide repeats ≥15 bases, tetranucleotide repeats ≥20 bases, pentanucleotide repeats ≥20 bases, and hexanucleotide or greater repeats ≥24 bases.

### PCR Amplification

To acquire a high quality complete chloroplast genome sequence, 129 primers (Table S1) were designed to increase the sequence accuracy by correcting 454 sequencing errors occurred in the homopolymer regions and to confirm the four junction regions between the IRs and SSC/LSC. PCR products were sequenced using BigDye V3.1 Terminator kit for ABI3730XL (Applied Biosystems, Foster City CA) and assembled into the complete chloroplast genome sequence using CONSED software.

To confirm the assembly accuracy at the junction regions of IRb with SSC and SSC with IRa in the *Artemisia frigida* cp genome, four primers (Table S1) was designed for the junction of IRb/SSC and SSC/IRa in *Artemisia frigida*. These primers were also used to examine the junction regions in other accessions of *Artemisia frigida* originated from Mongolia (PI 639180) and United States (W6 30042 from Colorado and AG 258 from Alaska) (available at Germplasm Resources Information Network http://www.ars-grin.gov/). The same strategy was also used to examine the junctions in *Helianthus annuus* and *Lactuca sativa* based on the sequence NC_007977 and DQ383816, respectively. The accession of HA410 for *Helianthus annuus* and the accession of LS01 for *Lactuca sativa* were used as template for PCR analyses. For PCR, each 20 µL PCR reaction system included 1× Gotaq buffer, 0.25 mM dNTP, 4 µM primers, 1 unit of homemade Taq polymerase, 6% DMSO, and 20 ng of DNA. The PCR amplification reactions were performed with 35 cycles of 50 sec denaturation at 94°C, 50 sec annealing at 52°C, and 90 sec extension at 72°C. PCR products were separated by electrophoresis in 1.5% agarose gel.

### Phylogenetic Analysis

A set of 61 protein-coding genes which have been analyzed in other species [48]–[50] were used to infer the phylogenetic relationships among *Artemisia frigida*, 56 angiosperm lineages previously published in the GenBank database, and 2 gymnosperms, *Pinus thunbergii* and *Ginko biloba* (Table S2). Sequences were aligned using ClustalW in MEGA5 [51], the alignment was edited manually. Phylogenetic analyses using maximum parsimony (MP) and maximum likelihood (ML) were performed with MEGA5 and the parameters were the same as Young described [52]. The high sequence diversity region found in the *ndhF* gene and the *trnL-trnF* region [53] were utilized for phylogenetic analyses among Asteraceae species. Both *ndhF* and *trnL-trnF* sequences of 92 species were downloaded from GenBank (Table S3). The concatenated sequence of *ndhF* and *trnL-F* were aligned using MUSCLE version 3.8 [54]. Maximum parsimony (MP) and maximum likelihood (ML) trees were reconstructed using above parameters with MEGA5. The gaps in the sequence alignment were treated as missing data.

## Results and Discussion

### Chloroplast Genome Assembly and Validation

One sequencing run of *Artemisia frigida* genomic DNA was carried out using Roche 454 sequencing technology on the GS FLX+ system. A total of 645,965 quality-filtered sequence reads were generated with the average read length of 598 bp, representing 387 Mb sequence data. Assembly of the nucleotide sequence reads was performed to obtain non-redundant contigs and singletons using the GS *De Novo* Assembler. In total, 28,129 contigs were assembled with a N50 contig size of 910 bp and a total accumulated length of 15,021,516 bp, representing only 0.15× coverage of the *Artemisia frigida* nuclear genome (2,567 Mb). The resulting contigs were searched against NCBI GenBank chloroplast genome database using BlastN and BlastX. Five contigs, with nucleotide length of 43,781 bp, 37,022 bp, 24,972 bp, 18,397 bp, and 1,937 bp were identified to be part of the cp genome. The number of sequence reads that were assembled into these five contigs were 4,465 (0.69% of the total 454 sequence reads) with an average read length of 638 bp. CONSED was used to reassemble these sequence reads extracted from the 454 sequence dataset. With the involvement of manual editing, a single sequence contig representing the entire *Artemisia frigida* cp genome was achieved. The average sequence depth of each nucleotide on the *Artemisia frigida* cp genome was 17.67×. The high sequence coverage from 454 reads allows for generation of consensus sequence with high accuracy.

Traditionally, sequencing of chloroplast genomes involved chloroplast isolation followed by purification of its DNA for library construction and sequencing [41]. Recently, several chloroplast genomes have been sequenced from nuclear genomic DNA with the use of high-throughput sequence systems such as SOLiD [55], Illumina [56], and 454 GS FLX platforms [30], [32], [57]. The chloroplast genomes are present in a high copy number in a single cell and often co-purified with nuclear genomic DNA as by-product or contamination. Because of their relative small genome sizes, the low percentage of chloroplast DNA sequence reads from the total nuclear genomic sequences generated by the next-generation high-throughput sequencing technologies can provided sufficient coverage for the assembly of chloroplast genomes [57]. Compared with the sequence read length generated by Illumina (∼150 bp) and SOLid (∼50 bp) sequencing methods, 454 GS FLX can generate longer sequence reads (∼400 bp). In general, longer reads will provide better sequence assembly at the same or similar sequence coverage, particularly for complex genomes with high repeat contents [57]. In our study, we used the 454 GS FLX+ platform which produced an average read length of 638 bp for the *Artemisia frigida* cp genome sequence reads. In the previous reports of cp genome sequencing by Roche 454, the average read length of mungbean, date palm and *Boea hygrometrica* are 217 bp [30], 347 bp [32], and 339 bp [57], respectively. Therefore, the sequence read length for the *Artemisia frigida* cp genome is more than 300 bp longer than that for these three cp genomes. However, the percentage of reads representing chloroplast DNA for *Artemisia frigida* (0.69%) is lower than mungbean (5.22%) [30], date palm (8.8%) [32], and *Boea hygrometrica* (0.91%) [57]. In our study, 387 Mb sequences representing 0.15× coverage of the *Artemisia frigida* genome (2,567 Mb) had enough chloroplast reads to assemble its entire cp genome, while 1× genome coverage (300 Mb) are required for the complete assembly of the cp genome in *Boea hygrometrica*. Our results indicated that sequence reads generated by 454 GS FLX+ platforms may be a better choice for *de novo* sequencing and assembly of organelle genomes since it can produce longer reads and make assembly easier and more robust.

The homopolymer issues in the 454 sequencing method usually cannot be overcome by increasing the coverage of the sequence data [30]–[32], [44]. To provide an accurate sequence for the *Artemisia frigida* chloroplast genome, resequencing of homopolymer regions by Sanger sequencing method was performed to determine the exact homopolymer lengths. PCR primer pairs (Table S1) were designed to cover 125 homopolymer regions (n >7 bp) based on the sequence of the initial *Artemisia frigida* cp genome assembly. Most of these homopolymer regions occurred in the non-coding regions. The results from resequencing of homopolymer regions showed that 29 base pairs were added or excluded in 125 homopolymers. This final *Artemisia frigida* cp genome sequence has been submitted to GenBank (GenBank ID: JX293720).

**Figure 1:**
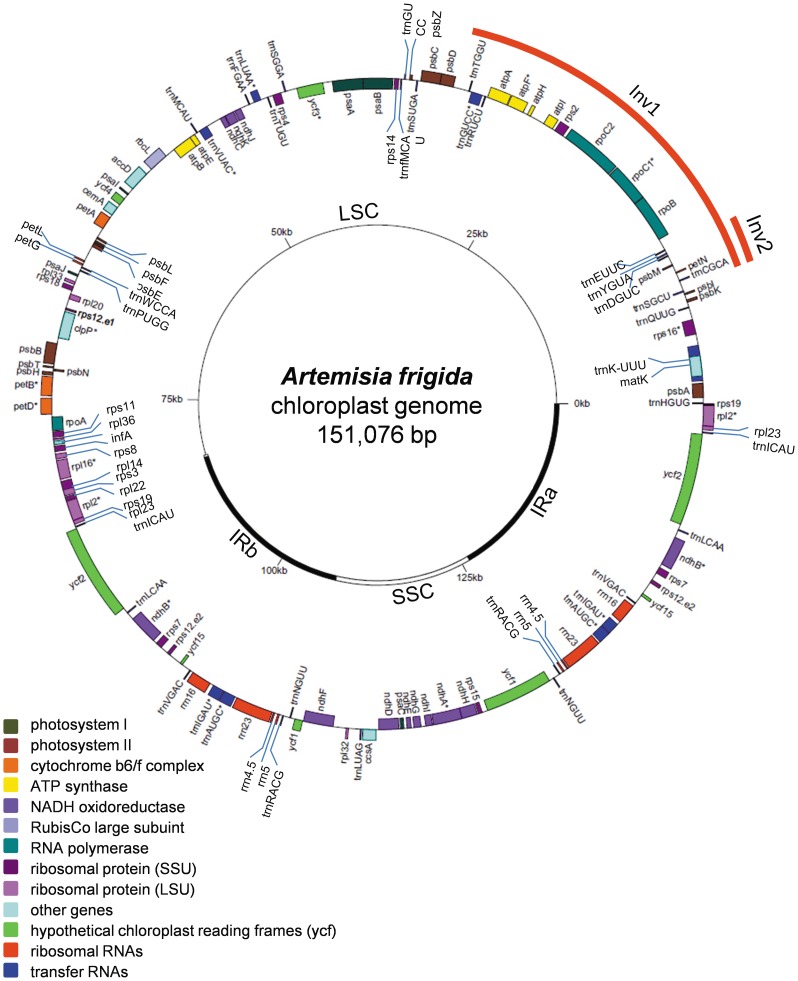
The map of the chloroplast genome of *Artemisia frigida*. IR, inverted repeat; LCS, large single copy region; SSC, small single copy region; Inv1, inverted sequence region 1; Inv2, inverted sequence region 2. Genes containing introns are marked with *.

The complete cp genome size of *Artemisia frigida* is 151,076 bp, including the LSC of 82,740 bp, the SSC of 18,394 bp and a pair of IRs of 24,971 bp each (Figure 1). The IRs span from *rpl2* to a portion of *ycf1*. The average AT content of the *Artemisia frigida* cp genome is 62.52%, which is consistent with the AT content reported for other plant cp genomes [41]. The AT contents of the LSC and SSC regions are 64.42% and 69.17%, respectively, whereas that of the IR regions is 56.93%.

### Genome Organization and Gene Content

The *Artemisia frigida* cp genome contains 114 unique genes, including 30 tRNA genes, 4 rRNA genes, and 80 predicted protein-coding genes (Table 1). In addition, there are 18 genes duplicated in the IR, making a total of 132 genes present in the *Artemisia frigida* cp genome (Figure 1). Protein-coding genes, tRNAs, and rRNAs make up 52.08%, 1.85%, and 5.99% of the genome, respectively, while the remaining 40.08% are non-coding introns, intergenic spacers, and pseudo genes. There are 18 intron-containing genes, including 6 tRNA genes and 12 protein-coding genes, almost all of which are single-intron genes except for *ycf3* and *clpP*, each having two introns. The *trnK*-UUU gene has the largest intron (2,564 bp) where another gene, *matK*, is located in it. We found that the two *rps12* genes, one in each IR region, are trans-spliced, with one of its exons located in the LSC (5′) and the other exon in the IR regions. Among the three pseudo genes, *ycf68* in the IR become pseudogenization due to several premature stop codons present in its coding sequence (Figure 1). Another two pseudo genes, *ycf1* and *rps19*, are located in the boundary regions between IRb/SSC and IRa/LSC, respectively. Incomplete duplication of the normal copy of *ycf1* and *rps19* at these boundaries has resulted in a lack of protein-coding ability.

**Table 1:** Genes present in *Artemisia frigida* chloroplast genome.

No.	Gene types	Gene products
1	photosystem I	psaA, B, C, I, J, ycf3^a^, ycf4
2	photosystem II	psbA, B, C,D, E, F, H, I, J, K, L, M, N, T, Z
3	Cytochrome b6/f	petA, B^b^, D^b^, G, L, N
4	Atp synthase	atpA, B, E, F^b^, H, I
5	Rubisco	rbcL
6	NADH oxidoreductase	ndhA^b^, Bb,c, C, D, E, F, G, H, I, J, K
7	Large subunit ribosomal proteins	rpl2b,c, 14, 16^b^, 20, 22, 23^c^, 32, 33, 36
8	Small subunit ribosomal proteins	rps2, 3, 4, 7^c^, 8, 11, 12b,c,d, 14, 15, 16^b^, 18, 19
9	RNAP	rpoA, B, C1^b^, C2
10	Other proteins	accD, ccsA, cemA, clpP^a^, matK, infA
11	Proteins of unknown fuction	ycf1, 2^c^, ycf15 c
12	Ribosomal RNAs	rrn16, 23, 4.5, 5
13	Transfer RNAs	trnA(UGC)b,c, C(GCA), D(GUC), E(UUC), F(GAA), G(UCC)^b^, G(UCC), H(GUG), I(CAU)^c^, I(GAU)b,c, K(UUU)^b^, L(CAA)^c^, L(UAA)^b^, L(UAG), fM(CAU), M(CAU), N(GUU)^c^, P(UGG), Q(UUG), R(ACG)^c^, R(UCU), S(GCU), S(GGA), S(UGA), T(GGU), T(UGU), V(GAC)^c^, V(UAC)^b^, W(CCA), Y(GUA)

a Gene containing two introns.

b Gene containing a single intron.

c Two gene copies in IRs.

d Gene divided into two independent transcription units.

Instead of a common ATG start codon, we identified two instances where ACG is used as a start codon: in *ndhD* and *psbL.* In addition, one GUG start codon is found in *rps19*. The ACG start codon has been shown to convert to an AUG initiation site as reported in *Nicotiana tabacum* [58]. Such RNA editing in the translation process likely also occurs in the *Artemisia frigida* cp genome.

There are 30 unique tRNA genes (7 tRNA genes duplicated in the IR) including two *trnG-UCC* genes in LSC region because of one with intron. These tRNA genes represented 20 amino acids identified in the cp genome (Table S2). A total of 26,226 codons represent the coding capacity of 86 protein-coding genes in the *Artemisia frigida* cp genome (Table S2). Isoleucine (2,208, 8.42%) and cysteine (288, 1.10%) are the most and the least abundant amino acids, respectively.

The cp genome size of *Artemisia frigida* is the third smallest among the seven completed Asteraceae cp genomes (after including *Artemisia frigida*). It is larger than *Jacobaea vulgaris* (150,689 bp) and *Ageratina adenophora* (150,698 bp) (Table S3), but smaller than the cp genomes of *Lactuca sativa*, *Helianthus annuus, Guizotia abyssinica, Parthenium argentatum* by 1.70 kb, 28 bp, 0.69 kb, and 1.73 kb, respectively. *Artemisia frigida* has the smallest LSC region (82,740 bp) among these sequenced Asteraceae cp genomes. The next smallest LSC region is from *Jacobaea vulgaris*, with a size of 82,855 bp.

**Figure 2:**
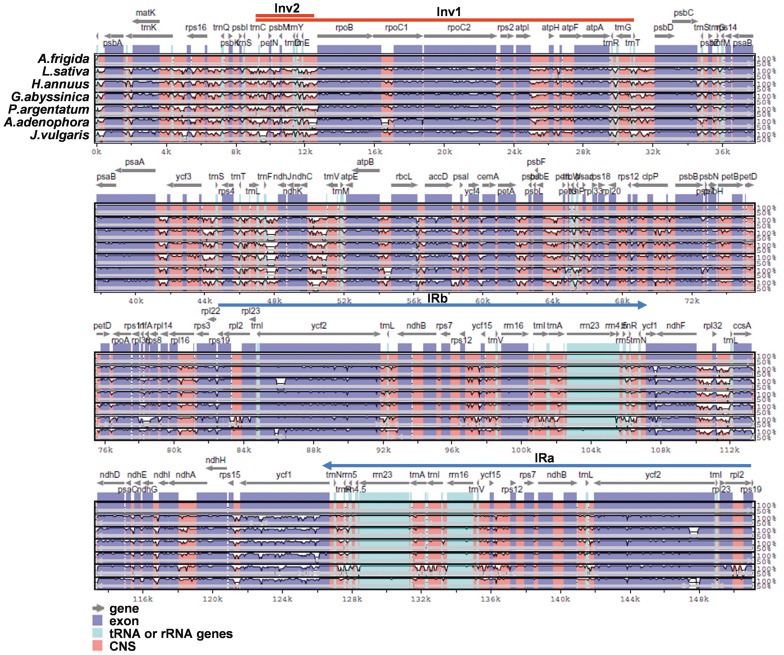
Sequence alignment of seven sequenced cp genomes in the Asteraceae family. Sequences of chloroplast genomes were aligned and compared using mVISTA program. A cut-off of 70% identity was used for the plot and the Y-scale represents the percent identity ranging from 50–100%. Blue represents exons, green-blue represents tRNA and rRNA genes, and pink represents conserved non-coding sequences (CNS). Grey arrows the direction of transcription; horizontal blue lines indicate the position of IRa and IRb; horizontal red lines indicate the position of Inv1 and Inv2.

Although chloroplast genomes are considered highly conserved among land plants, regions with highly sequence polymorphisms were often observed even among closely related species [59]. Alignments of seven sequenced cp genome sequences available in the Asteraceae family were performed using mVISTA program family with the new annotation of *Artemisia frigida* to reveal their sequence variations. This analysis showed that the coding region is more conserved than the non-coding region, and that the most divergent coding regions in the seven genomes were *ycf1*, *accD*, *ccsA*, *rps16* and *rpoC1* (Figure 2).

In addition to the various nucleotide divergence in different regions, sequence arrangements also occurred in cp genomes. Comparing with the cp genome of *Nicotiana tabacum,* the cp genome of *Artemisia frigida* had two inversion events in the LSC region. The sizes of the two inversions were 22,837 bp (Inv1) and 3,421 bp (Inv2). The large inversion (Inv1) changed the order genes located in this inversion region as compared to that in *Nicotiana tabacum* (Figure 1 and Figure 2). The second small inversion (Inv2) is within the region of the large inversion. Both inversions started at the position of 8,837 bp while Inv2 ended at 12, 257 bp and Inv1at 31,674 bp. It appears that the two inversions occurred within the same evolutionary time period as what existed in most of the Asteraceae family, including *Lactuca sativa*, *Helianthus annuus, Guizotia abyssinica, Parthenium argentatum, Ageratina adenophora,* and *Jacobaea vulgaris* [38]–[42], [60] (Figure 1 and Figure 2).

**Figure 3:**
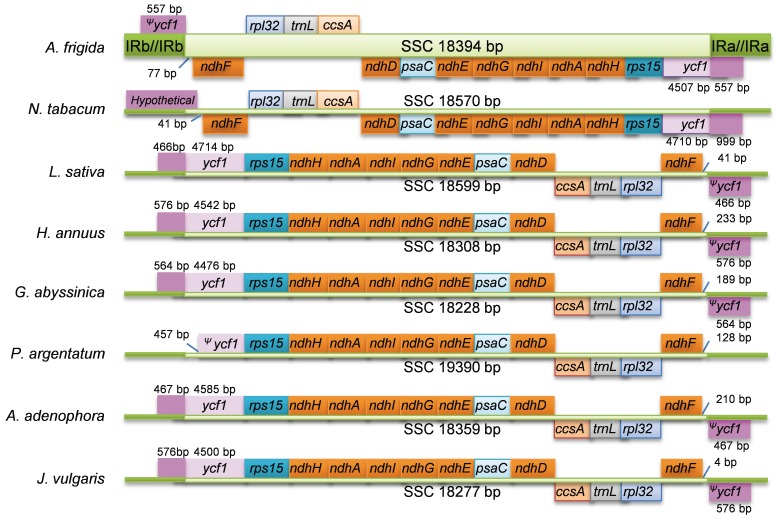
Comparison of the SSC region among different Asteraceae species. Gene sequences in the SSR region were annotated and indicated along the green lines. Genes above the green lines indicate their transcriptions in forward direction and genes below the green lines represent their transcriptions in reverse direction.

We also analyzed the gene order in the SSC region. The tobacco cp genome is often regarded to be unaltered [22] and therefore used as reference here (Figure 3). The gene order in the SSC region in tobacco and *Artemisia frigida* begins with *ndhF*, and then is followed by the order of *rpl32, trnL, ccsA, ndhD, psaC, ndhE, ndhG, ndhI, ndhA, ndhH and rps15,* and ends with *ycf1,* which is extended into IRa regions. The gene orders of the other 6 species in the Asteraceae family are the completely same, but inverted compared to *Artemisia frigida*. Given the notion that most species in the Asteraceae family have the same gene order in the SSC region, it is likely that an inversion in the SSC region occurred before the divergence of species in the Asteraceae family. The fact that *Artemisia frigida* has the same gene order in the SSC region with *Nicotiana tabacum* suggests that re-inversion in the SSC region occurred in *Artemisia frigida* lineage.

**Figure 4:**
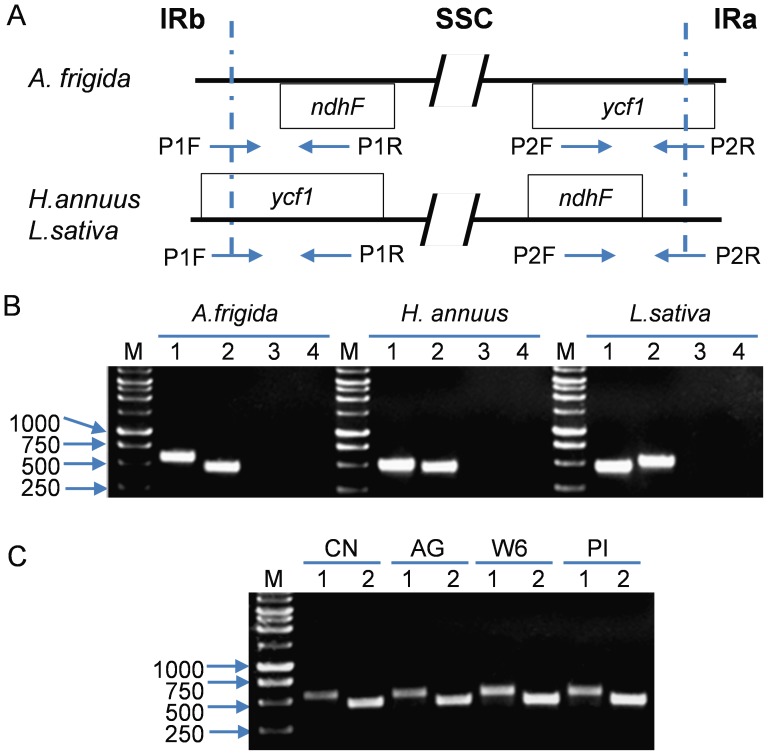
Analyses of SSC inversions in the Asteraceae family. **A.** Primer design to amplify junction regions between IR and SSC regions. The positions of *ndhF* and *ycf1* genes in relation to IRa and IRb regions are drawn based on the sequence assembly results of *A. frigida* in this study and *H. annuus* and *L. sativa* from published data [38]. To design primers that amplify the junction region between IRa and left border of the SSC, the forward primer P1F contains the sequence with half of length from IRb and the other half from the SSC region. The sequence of the reverse primer (P1R) is located in the *ndhF* gene. The same strategy was employed in primer design to amplify the other junction region between *ycf1* and IRa regions as indicated. The same strategy was also used to examine the junctions in *H. annuus* and *L. sativa* based on their assembled sequences as indicated in the diagram. **B.** PCR amplification of IR and SSC junction regions in *Artemisia frigida, Helianthus annuus* and *Lactuca sativa.* M:Promega 1 kb ladder; lane 1, primer pair P1F and P1R, lane 2, primer pair P2F and P2R, lane 3, primer pair P1F and P2F, and lane 4, primer pair P1R and P2R. **C.** PCR amplification of IR and SSC junction regions in different accessions of *Artemisia frigida*. Four *Artemisia frigida* accessions were used in PCR reactions; CN: cp sequenced accession in this study and originated from China, AG:AG258 from Alaska, USA, W6: W6 30042 from Colorado, USA, and PI: PI 639180 from Mongolia. Lane 1, primer pair P1F and P1R, Lane 2, primer pair P2F and P2R.

To further confirm that the gene order in the SSC in *Artemisia frigida* is different from those in the Asteraceae family, four primers were designed for each species to amplify the junctions of IRb/SSC and SSC/IRa in *Artemisia frigida*, *Helianthus annuus*, *Lactuca sativa* and from different accessions of *Artemisia frigida* (Figure 4A). The primer pairs of P1F/P1R and P2F/P2R amplified PCR products in *Artemisia frigida* while the primer combinations of P1F/P2F and P1R/P2R had no PCR products (Figure 4B). In two other species in the Asteraceae family, HA410 (*Helianthus annuus*) and LS01 (*Lactuca sativa*) provided amplified PCR products using the primer pairs of P1F/P1R and P2F/P2R. No PCR products were amplified with the primer pairs of P1F/P2F and P1R/P2R (Figure 4B). These results indicated that the SSC region in *Artemisia frigida* is re-inverted comparing to *Helianthus annuus* and *Lactuca sativa*. We further examined this re-inversion event in other *Artemisia frigida* accessions collected from different geographical regions (PI 639180 from Mogolia, W6 30042 from Colorado, and AG 258 from Alaska). The results showed that these three accessions provided PCR products with the primer pairs of P1F/P1R and P2F/P2R (Figure 4C), indicating that they have the same gene order with the sequenced *Artemisia frigida* accession. It is likely that these accessions in *Artemisia frigida* shared the same re-inversion event.

The identification and characterization of inversion and re-inversion events in *Artemisia frigida* suggests that the SSC might be an active region for sequence rearrangements in plant cp genomes. We therefore searched the SSC regions of sequenced cp genomes in plants. Most species share the same cpDNA organization in the SSC region with *Nicotiana tabacum* [22]. However, some angiosperm species such as *Piper cenocladum* (magnoliids) [44], *Dioscorea elephantipes* (Dioscoreaceae) (monocots) [45], and *Chloranthus spicatus* (Chloranthaceae) (basal angiosperm) [45] have an inverted SSC region (data not shown). Although chloroplast genomes are generally conserved in gene order in land plants [17], [61], several sequence rearrangements in cp genomes from different plant species have been reported, including a large inversion in LSC region [19]–[21], [62], IR contraction or expansions into single copy region with inversions [30], [63], and SSC region as shown in this study. It has been proposed that intramolecular recombination events are the causes of sequence rearrangements in the cp genomes [64], [65]. These sequence rearrangements that alter cp genome structures in related species could provide the genetic diversity useful for molecular classification and evolution studies.

### Repeat Sequence Analysis and Distribution of cp SSR

**Figure 5:**
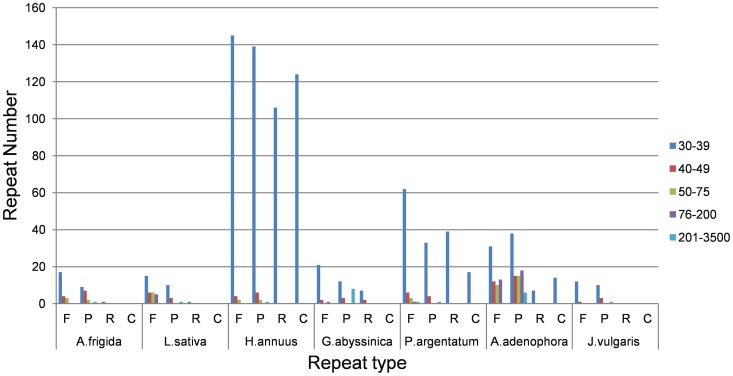
Repeat sequence analysis in seven sequenced Asteraceae chloroplast genomes. REPuter was used to identify repeat sequences with length ≥30 bp long and sequence identity ≥90% in the cp genomes. F, P, R, and C indicate that the repeats matching in forward, palindrome, reverse, and complement orientations, respectively. Different repeat unit lengths are indicted with different color.

We used REPuter to analyze the repeat sequences in the *Artemisia frigida* cp genome and found 24 direct (forward) repeats, 18 inverted (palindrome) repeats, and 1 reverse repeat of at least 30 bp long per repeat unit with a sequence identity of 90% and above (Table 2). Twenty-seven repeats are 30–40 bp long, 11 repeats are 41–50 bp long, and 5 repeats are 51–60 bp long. The repeat structures of the other six species within Asteraceae were also analyzed by REPuter (Figure 5). Forward repeats and inverted repeats are common in these species. The repeat structure of *Artemisia frigida,* which is from the Anthemideae tribe, is similar to those of *Lactuca sativa, Guizotia abyssinica,* and *Jacobaea vulgaris,* which are from the Cichorieae, Heliantheae alliance, and Senecioneae tribe, respectively. *Helianthus annuus*, *Parthenium argentatum,* and *Ageratina adenophora* are all in the same Heliantheae alliance tribe, but the repeat structures of these species are different. The *Helianthus annuus* cp genome contains the greatest number and variety of repeats, while *Parthenium argentatum* shares the same repeat structure, but has fewer overall repeats. Of the 7 Asteraceae cp genomes studied, *Ageratina adenophora* contains the greatest total number of repeats that are 40 bp or greater in length. The reason for this may be because of the different subtribe and genus to which *Ageratina adenophora* belongs.

**Table 2:** Repeat sequences in the *Artemisia frigida* chloroplast genome.

Repeat No.	Repeat size (bp)	Repeat 1 start	Repeat 2 start	Repeat type	Repeat 1 location	Repeat 2 location
1	33	5019	5034	F	IGS *(trnK-UUU- rps16)*	IGS *(trnK-UUU - rps16)*
2	32	8441	34788	F	I *trnS-GCU*	I *trnS-UGA*
3	30	27926	29521	F	IGS *(atpF - atpA)*	IGS *(atpA - trnR-UCU)*
4	32	37989	40213	F	*psaB*	*psaA*
5	30	38000	40224	F	*psaB*	*psaA*
6	41	42994	96732	F	Intron *(ycf3)*	IGS *(rps7 - ycf15)*
7	39	42996	118042	F	Intron *(ycf3)*	Intron *(ndhA)*
8	35	42999	93683	F	Intron *(ycf3)*	Intron *(ndhB)*
9	30	56190	56214	F	I *rbcL*	IGS *(rbcL -accD)*
10	30	66201	97916	F	IGS *(psaJ - rpl33)*	IGS *(ycf15 - trnV-GAC)*
11	30	86397	147393	F	*ycf2*	*ycf2*
12	60	89967	89985	F	*ycf2*	*ycf2*
13	42	89967	90003	F	*ycf2*	*ycf2*
14	30	89979	90015	F	*ycf2*	*ycf2*
15	45	89982	90000	F	*ycf2*	*ycf2*
16	39	96734	118042	F	IGS *(rps7 - ycf15)*	Intron *(ndhA)*
17	30	105646	105678	F	IGS *(rrn4.5 - rrn5)*	IGS *(rrn4.5 - rrn5)*
18	30	121294	121295	F	IGS *(rps15 - ycf1)*	IGS *(rps15 - ycf1)*
19	36	122447	122777	F	*ycf1*	*ycf1*
20	30	128108	128140	F	IGS *(rrn4.5 - rrn5)*	IGS *(rrn4.5 - rrn5)*
21	60	143771	143789	F	*ycf2*	*ycf2*
22	42	143771	143807	F	*ycf2*	*ycf2*
23	52	143779	143797	F	*ycf2*	*ycf2*
24	34	143779	143815	F	*ycf2*	*ycf2*
25	30	8443	44679	P	I *trnS-GCU*	I *trnS-UGA*
26	30	34790	44679	P	I *trnS-UGA*	I *trnS-UGA*
27	41	42994	137043	P	Intron *(ycf3)*	IGS *(ycf15-rps7)*
28	35	42999	140098	P	Intron *(ycf3)*	Intron *(ndhB)*
29	30	66201	135870	P	IGS *(psaJ - rpl33)*	IGS *(trnV-GAC-ycf15)*
30	48	72915	72915	P	IGS *(psbT -psbN)*	IGS *(psbT -psbN)*
31	60	89967	143771	P	*ycf2*	*ycf2*
32	42	89967	143771	P	*ycf2*	*ycf2*
33	30	89979	143771	P	*ycf2*	*ycf2*
34	45	89982	143771	P	*ycf2*	*ycf2*
35	60	89985	143789	P	*ycf2*	*ycf2*
36	45	90000	143789	P	*ycf2*	*ycf2*
37	42	90003	143807	P	*ycf2*	*ycf2*
38	30	90015	143807	P	*ycf2*	*ycf2*
39	30	105646	128108	P	IGS *(rrn4.5 - rrn5)*	IGS *(rrn4.5 - rrn5)*
40	30	105678	128140	P	IGS *(rrn4.5 - rrn5)*	IGS *(rrn4.5 - rrn5)*
41	43	114927	114927	P	IGS *(ndhD - psaC)*	IGS *(ndhD - psaC)*
42	39	118042	137043	P	Intron *(ndhA)*	IGS *(ycf15-rps7)*
43	31	121291	121291	R	IGS *(rps15 - ycf1)*	IGS *(rps15 - ycf1)*

I partly in the IGS region; F-forward, P-palindrome, R-reverse, IGS-Intergenic spacer region.

**Figure 6:**
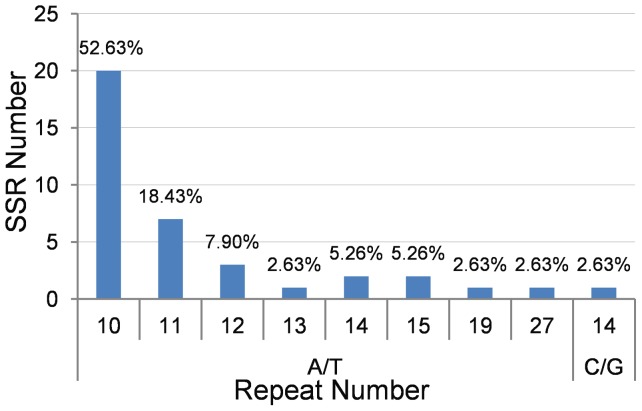
The distribution and frequency of simple sequence repeats in the *Artemisia frigida* cp genome. The SSR length is implied by the number of repeat for each SSR type in the Y-axes The percentage of a repeat with specific length from the total number of repeats is indicated above the bars.

Another type of repeat sequences frequently occurred in the cp genomes is the simple sequence repeats (SSRs). The distribution of SSRs was analyzed for the *Artemisia frigida* cp genome. Thirty-eight (38) mononucleotide SSRs (92.68%), also called homopolymers, 2 dinucleotide SSRs (4.88%), and 1 trinucleotide SSR (2.44%) were identified (Table 3). Thirty-three (33) of the 41 SSR loci were found in the intergenic regions, 3 were located in introns, and the other 5 SSRs were located in genes. Among the 38 mononucleotide SSRs, only one C/G type was found, while all others belonged to the A/T type. The repeat number of mononucleotide motifs ranged from 10 to 27, and 52.63% of the repeats were A/T type with repeat number 10 (Figure 6).

**Table 3:** Simple sequence repeat in *Artemisia frigida* chloroplast genome.

No.	location	SSR type	SSR start	SSR end
1	*psbA-trnK-UUU*	(A/T)10	1674	1683
2	*trnK-UUU-matK*	(A/T)15	1957	1971
3	*trnK-UUU-rps16*	(A/T)10	4733	4742
4	*rps16* intron	(C/G)14	5430	5443
5	*psbM-trnD-GUC*	(A/T)10	11206	11215
6	*trnE-UUC-rpoB*	(A/T)15	12645	12659
7	*rpoB*	(A/T)10	13335	13344
8	*rpoC1* intron	(A/T)10	16604	16613
9	*rpoC1*	(A/T)10	17957	17966
10	*rpoC1*	(A/T)10	18383	18392
11	*rpoC2-rps2*	(A/T)12	23084	23095
12	*atpI-atpH*	(A/T)10	26017	26026
13	*atpF-atpA*	(A/T)12	27936	27947
14	*atpA-trnR-UCU*	(A/T)11	29531	29541
15	*trnR-UCU-trnG-UCC*	(A/T)12	29749	29760
16	*psbC-trnS-UGA*	(A/T)19	34644	34662
17	*psbZ-trnG*	(A/T)10	35513	35522
18	*psaA-ycf3*	(A/T)13	41570	41582
19	*psaA-ycf3*	(A/T)10	41754	41763
20	*ndhC-trnV-UAC*	(A/T)10	50173	50182
21	*atpB-rbcL*	(A/T)14	54366	54379
22	*rbcL-accD*	(A/T)10	56590	56599
23	*petA-psbJ*	(A/T)11	62051	62061
24	*psbE-petL*	(A/T)10	64272	64281
25	*rps18-rpl20*	(A/T)11	67100	67110
26	*clpP* intron	(A/T)11	69197	69207
27	*rpoA*	(A/T)10	76711	76720
28	*rps8-rpl14*	(A/T)14	79128	79141
29	*rpl14-rpl16*	(A/T)10	79631	79640
30	*rrn5-trnR-ACG*	(A/T)11	106007	106017
31	*trnR-ACG-trnN-GUU*	(A/T)10	106475	106484
32	*ndhF-rpl32*	(A/T)10	110893	110902
33	*rpl32-trnL-UAG*	(A/T)10	111959	111968
34	*ndhD-psaC*	(A/T)10	114977	114986
35	*rps15-ycf1*	(A/T)27	121295	121321
36	*rps15-ycf1*	(A/T)11	121534	121544
37	*trnN-GUU-trnR-ACG*	(A/T)10	127333	127342
38	*trnR-ACG-rrn5*	(A/T)11	127800	127810
39	*petN-psbM*	(AT)6	10217	10228
40	*trnT-GGU-psbD*	(TA)6	30963	30974
41	*ycf1*	(TTC)5	125892	125906

Chloroplast SSRs (cpSSRs) are generally short mononucleotide tandem repeats that, when located in the noncoding regions of the cp genome, commonly show intraspecific variation in repeat number [66], [67]. In our study, 34 of 38 mononucleotide SSR loci (≥10 bases) occurred in nocoding regions, including 31 in the intergenic regions and 3 in introns (Table 2). Compared with other species of angiosperms, the number of mononucleotide cpSSR in *Artemisia frigida* found in non-coding regions of the cp genome was much greater. Several species contain less than 34 mononucleotide cpSSRs in non-coding regions, including *Helianthus annuus* (Asteraceae) (30), *Panax ginseng* (Araliaceae) (9), *Daucus carota* (Apiaceae) (23), 7 species from three genera in Solanaceae (28–31), 5 species from two genera in Convolvulaceae (12–33), as well as other species [68]. However, *Artemisia frigida* also contains less non-coding mononucleotide cpSSRs than *Cucumis sativus* (Cucurbitaceae) (47), *Citrus sinensis* (Rutaceae) (60), *Vitis vinifera* (Vitaceae) (46), and other species [68]. Like other chloroplast markers which are uniparental in inheritance, cpSSRs have been widely used in the analysis of plant population structure, diversity, differentiation and maternity analysis. Inter- and intra-specific chloroplast variation has also been studied within plant populations, including many species of Poaceae [69]–[71], Solanaceae [72], and Brassicaceae [73], [74]. While the applicable use of cpSSR is still largely centered on economically important plants and their relatives, the potential for cpSSRs to offer unique insights into ecological and evolutionary processes in wild plant species is quite substantial and not yet fully realized [68]. Our results provide cpSSR markers for the analysis of genetic diversity in *Artemisia frigida* and its relative species and provide an efficient means to select germplasm with high pharmaceutical efficacy.

### Phylogenetic Analysis

**Figure 7:**
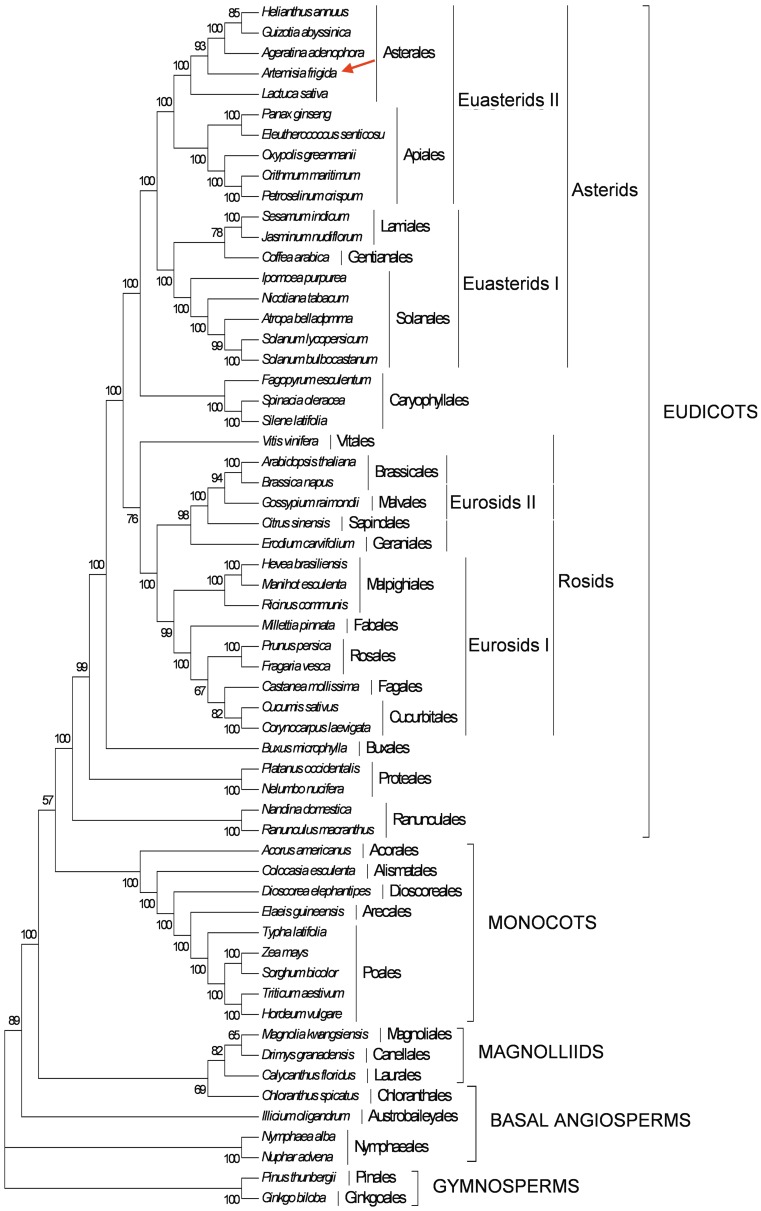
Phylogenetic tree reconstruction of 59 taxa using maximum likelihood (ML) based on concatenated sequence from 61 chloroplast protein-coding genes. The position of *Artemisia frigida* is indicated by a red arrow.

*Artemisia frigida* belongs to the tribe Anthemideae in the Asteraceae family. Several studies have been conducted to analyze the phylogenetic relationship in the Asteraceae family based on chloroplast coding or non-coding sequences [53], [75], [76]. The phylogenetic evolution of *Artemisia frigida* has only been studied by using *trnSUGA-trnfMCAU*, *trnSGCU-trnCGCA* [13], *psbA-trnH*, *rpl32-trnL* [16], and nucleic DNA sequence 3-ETS, ITS [11] within the genus *Artemisia L*. The chloroplast gene *ndhF* has been used successfully to conduct phylogenies at the intergenetic and interfamilial levels within Asteraceae [59], Bromeliaceae [77], and Acanthaceae [78], among others [79]. The *trnL-F* non-coding region has been widely used for reconstructing phylogenies between closely related species and for identifying plant species [80]–[82]. Many uncertainties are still remaining in the molecular phylogeny of the Asteraceae family and molecular evidence to support the phylogenetic position of *Artemisia frigida* is still lacking. The availability of completed *Artemisia frigida* cp genome provided us with the sequence information to study the molecular evolution and phylogeny of *Artemisia frigida* with closely related species. We first extracted 61 protein-coding genes from sequenced cp genomes from species belonging to 59 taxa, including 5 Asteraceae species (Table S4). After sequence alignment, all positions containing gaps and missing data were eliminated, leaving a total of 39,140 positions in the final dataset. ML analysis based on the Tamura-Nei model [83] resulted in a single tree with ln L = −451091.42 (Figure 7). Bootstrap analysis indicated that 44 of 55 nodes were supported by values ≥95% and 40 of these with bootstrap values of 100%. MP analysis resulted in a single tree with a length of 81, 210, a consistency index of 0.3447, and a retention index of 0.5978 (data not shown). The ML and MP trees had similar phylogenetic topologies. *Artemisia frigida* grouped together with *Helianthus annuus*, *Guizotia abyssinica,* and *Ageratina adenophora* in the supertribe Helianthodae, all within the subfamily Asteroideae. *Lactuca sativa* was grouped with the tribe Lactuceae of another subfamily, Cichorioideae, within the Asteraceae. The five species in the Asteraceae family were clustered into Asterales and placed within the euasterids II. In addition, the tribe Anthemideae demonstrates a closer relationship with the tribe Heliantheae than with Lactuceae. Through our analysis it was determined that *Cucumis*, whose phylogenetic position was not yet completely determined [84], was grouped within the eurosids I clade, which is comparable to the result of Nie et al. [41].

Further phylogenetic analysis was performed using *ndhF* and *trnL-F* sequences on 90 species in the Asteraceae family including *Artemisia frigida* (Table S5 and Figure S1). Both ML and MP trees were reconstructed for placement of phylogenic positions of these selected species. There were a total of 2,417 nucleotide alignment sites in the final dataset for the tree reconstructions. ML analysis based on the Tamura-Nei model [83] generated a single tree with ln L = −20049.26 (Figure S1). MP analysis resulted in a single tree with a length of 2,625, a consistency index of 0.4388, and a retention index of 0.6292 (data not shown). Both ML and MP trees provide strong support for *Artemisia frigida* being clustered into the Anthemideae tribe in the subfamily Asteroideae. As for the 6 species which have sequenced cp genomes, *Helianthus annuus, Parthenium argentatum, Ageratina adenophora*, and *Guizotia abyssinica* fall into the Heliantheae alliance tribe of Asteroideae, *Jacobaea vulgaris* is located in the Senecioneae tribe of Asteroideae, and *Lactuca sativa* is grouped into the Cichorieae tribe in the Cichorioideae subfamily. Finally, *Artemisia frigida* grouped into the Anthemideae tribe has a closer relationship with the Heliantheae alliance and Senecioneae tribes than with Cichorieae in the phylogenetic tree (Figure S1). The phylogeny obtained with the molecular data is consistent with the classification based on phenotypic observation [85].

### Conclusions

Genomic DNA of *Artemisia frigida* was sequenced using 454 pyrosequencing technology and the complete chloroplast genome was identified and annotated. This is the first cp genome sequenced in the Anthemideae tribe within the Asteraceae family. We found that most Asteraceae species have an inverted SSC region in comparison with the unaltered tobacco cp genome. However, re-inversion event has occurred in the SSC region in *Artemisia frigida* lineage, suggesting that SSC might be an active region for inversion events. Repeat sequences were also analyzed in this study to explore the use of polymorphic microsatellites at the intra- and inter-specific level among *Artemisia* species. Sixty-one (61) protein-coding sequences from 59 species were employed to construct phylogenic trees, providing a strong support for a monophyletic group of the asteroids II clade. *Artemisia frigida* also demonstrated a close relationship to *Helianthus annuus, Guizotia abyssinica,* and *Ageratina adenophora,* which belong to the subfamily Asteroideae. In the Asteraceae family, *Artemisia frigida* clustered into the Anthemideae tribe in the subfamily Asteroideae based on *ndhF* and *trnL-F* gene sequence analysis. *Artemisia frigida* is the seventh cp genome of the Asteraceae family to be described. It will be useful for molecular ecology and molecular phylogeny studies within this species and also within the Asteraceae family.

## Acknowledgments

The authors would like to thank Professor Chunlin Long, Minzu University of China, for critical review of the manuscript. The authors would like to thank Zhao Liu, North Dakota State University, for providing HA410 genomic DNA for *Helianthus annuus*.

## Supporting Information

Figure S1 Reconstruction of phylogentic tree of Asteraceae and related families. The tree topology was constructed with the maximum likelihood method using the *ndhF* and *trnL-F* gene sequence regions. Bootstrp proportions shown above the branches. lnL = −20049.26. The position of the sequenced *Artemisia frigida* species is indicated with a red arrow.
(TIF)

Table S1 List of primer pairs used in sequence verification and improvement of the *Artemisia frigida* chloroplast genome.
(DOC)

Table S2 The codon-anticondon recognition pattern and codon usage for *Artemisia frigida* chloroplast genome.
(DOC)

Table S3 Size comparison of *Artemisia frigida* chloroplast genomic regions with those in other species of Asteraceae.
(DOC)

Table S4 The GenBank accession numbers of all the 58 cp genomes used for phylogenetic analysis.
(DOC)

Table S5 The GenBank accession numbers of *ndhF* gene and *trnL-F* gene region from all the 92 species used for phylogenetic analysis.
(DOC)

## References

[1] Liu Y, Liu J, Tang L, Zhang W, Zhang L, et al (2008) Current Status Research of Mongolian Medicine Artemisia Frigida Willd. Studies of Trace Elements and Health 25: 55–57.

[2] Bremer K (1994) Asteraceae - Cladistics and Classification. Portland, Oregon, USA: Timber Press.

[3] Garcia S, Sanz M, Garnatje T, Kreitschitz A, McArthur ED, et al (2004) Variation of DNA amount in 47 populations of the subtribe Artemisiinae and related taxa (Asteraceae, Anthemideae): karyological, ecological, and systematic implications. Genome 47: 1004–1014. 15644958 10.1139/g04-061

[4] Wan T, Sun Q, Cai P, Meng X, Yi W, et al (2011) Observation of chromosome karyotypes of Artemisia frigida in different ecological areas of Inner Mongolia. Acta Botanica Boreali-Occidentalia Sinica 31: 456–461.

[5] Tian YH (2000) Chinese national medicine processing integration. Beijing: Chines Ancient Books Press.

[6] Zhang W, Tang L, Xie K, Cui J (2008) Determination of total flavonoids in raw and charred Mongolian drug Agi. Li Shizhen Medicine and Materia Medica Research 19: 2952–2953.

[7] Cui J, Tang L, Lan R (2006) Baoyindalai (2006) Clinical analysis of 63 cases on curative effect of Arteisia frigida Wild in treatment of hemoptysis caused by bronchiectasis. Journal of Minzu University of China (natural sciences edition) 15: 149–155.

[8] Piao XL, Xie K, Cui J (2009) Fatty components of Artemisia frigida before and after carbonization by GC-MS. Li Shizhen Medicine and Materia Medica Research 20: 1848–1849.

[9] Wang QH, Ao WL, Wang XL, Bao XH, Wang JH (2010) Two new flavonoid glycosides from Artemisia frigida Willd. J Asian Nat Prod Res 12: 950–954. 21061216 10.1080/10286020.2010.510469

[10] Taberlet P, Gielly L, Pautou G, Bouvet J (1991) Universal primers for amplification of three non-coding regions of chloroplast DNA. Plant Mol Biol 17: 1105–1109. 1932684 10.1007/BF00037152

[11] Tkach NV, Hoffmann MH, Roser M, Korobkov AA, von Hagen KB (2008) Parallel evolutionary patterns in multiple lineages of arctic Artemisia L. (Asteraceae). Evolution 62: 184–198. 17976192 10.1111/j.1558-5646.2007.00270.x

[12] Soininen EM, Valentini A, Coissac E, Miquel C, Gielly L, et al (2009) Analysing diet of small herbivores: the efficiency of DNA barcoding coupled with high-throughput pyrosequencing for deciphering the composition of complex plant mixtures. Front Zool 6: 16. 19695081 10.1186/1742-9994-6-16 PMC2736939

[13] Garcia S, McArthur ED, Pellicer J, Sanderson SC, Valles J, et al (2011) A molecular phylogenetic approach to western North America endemic Artemisia and allies (Asteraceae): Untangling the sagebrushes. Am J Bot 98: 638–653. 21613164 10.3732/ajb.1000386

[14] Hiiesalu I, Opik M, Metsis M, Lilje L, Davison J, et al (2012) Plant species richness belowground: higher richness and new patterns revealed by next-generation sequencing. Mol Ecol 21: 2004–2016. 22168247 10.1111/j.1365-294X.2011.05390.x

[15] Frank DA, Pontes AW, Maine EM, Caruana J, Raina R, et al (2010) Grassland root communities: species distributions and how they are linked to aboveground abundance. Ecology 91: 3201–3209. 21141181 10.1890/09-1831.1

[16] Riggins CW, Seigler DS (2012) The genus Artemisia (Asteraceae: Anthemideae) at a continental crossroads: Molecular insights into migrations, disjunctions, and reticulations among Old and New World species from a Beringian perspective. Mol Phylogenet Evol 64: 471–490. 22580463 10.1016/j.ympev.2012.05.003

[17] Palmer JD (1991) Plastid chromosomes: structure and evolution; Bogorad L, Vasil IK, editors. San Diego: Academic Press.

[18] Chumley TW, Palmer JD, Mower JP, Fourcade HM, Calie PJ, et al (2006) The complete chloroplast genome sequence of Pelargonium x hortorum: organization and evolution of the largest and most highly rearranged chloroplast genome of land plants. Mol Biol Evol 23: 2175–2190. 16916942 10.1093/molbev/msl089

[19] Jansen RK, Palmer JD (1987) A chloroplast DNA inversion marks an ancient evolutionary split in the sunflower family (Asteraceae). Proc Natl Acad Sci U S A 84: 5818–5822. 16593871 10.1073/pnas.84.16.5818 PMC298954

[20] Doyle JJ, Doyle JL, Ballenger JA, Palmer JD (1996) The distribution and phylogenetic significance of a 50-kb chloroplast DNA inversion in the flowering plant family Leguminosae. Mol Phylogenet Evol 5: 429–438. 8728401 10.1006/mpev.1996.0038

[21] Doyle JJ, Davis JI, Soreng RJ, Garvin D, Anderson MJ (1992) Chloroplast DNA inversions and the origin of the grass family (Poaceae). Proc Natl Acad Sci U S A 89: 7722–7726. 1502190 10.1073/pnas.89.16.7722 PMC49783

[22] Shinozaki K, Ohme M, Tanaka M, Wakasugi T, Hayashida N, et al (1986) The complete nucleotide sequence of the tobacco chloroplast genome: its gene organization and expression. EMBO J 5: 2043–2049. 16453699 10.1002/j.1460-2075.1986.tb04464.x PMC1167080

[23] De Cosa B, Moar W, Lee SB, Miller M, Daniell H (2001) Overexpression of the Bt cry2Aa2 operon in chloroplasts leads to formation of insecticidal crystals. Nat Biotechnol 19: 71–74. 11135556 10.1038/83559 PMC4560096

[24] Ruiz ON, Hussein HS, Terry N, Daniell H (2003) Phytoremediation of organomercurial compounds via chloroplast genetic engineering. Plant Physiol 132: 1344–1352. 12857816 10.1104/pp.103.020958 PMC167074

[25] Quesada-Vargas T, Ruiz ON, Daniell H (2005) Characterization of heterologous multigene operons in transgenic chloroplasts: transcription, processing, and translation. Plant Physiol 138: 1746–1762. 15980187 10.1104/pp.105.063040 PMC1176443

[26] Hagemann R (2004) The sexual inheritance of plant organelles; Daniell H, Chase CD, editors: Springer, Dordrecht, The Netherlands.

[27] Dufourmantel N, Pelissier B, Garcon F, Peltier G, Ferullo JM, et al (2004) Generation of fertile transplastomic soybean. Plant Mol Biol 55: 479–489. 15604694 10.1007/s11103-004-0192-4

[28] Kumar S, Dhingra A, Daniell H (2004) Plastid-expressed betaine aldehyde dehydrogenase gene in carrot cultured cells, roots, and leaves confers enhanced salt tolerance. Plant Physiol 136: 2843–2854. 15347789 10.1104/pp.104.045187 PMC523346

[29] Kumar S, Dhingra A, Daniell H (2004) Stable transformation of the cotton plastid genome and maternal inheritance of transgenes. Plant Mol Biol 56: 203–216. 15604738 10.1007/s11103-004-2907-y PMC3481848

[30] Tangphatsornruang S, Sangsrakru D, Chanprasert J, Uthaipaisanwong P, Yoocha T, et al (2010) The chloroplast genome sequence of mungbean (Vigna radiata) determined by high-throughput pyrosequencing: structural organization and phylogenetic relationships. DNA Res 17: 11–22. 20007682 10.1093/dnares/dsp025 PMC2818187

[31] Moore MJ, Dhingra A, Soltis PS, Shaw R, Farmerie WG, et al (2006) Rapid and accurate pyrosequencing of angiosperm plastid genomes. BMC Plant Biol 6: 17. 16934154 10.1186/1471-2229-6-17 PMC1564139

[32] Yang M, Zhang X, Liu G, Yin Y, Chen K, et al (2010) The complete chloroplast genome sequence of date palm (Phoenix dactylifera L.). PLoS One 5: e12762. 20856810 10.1371/journal.pone.0012762 PMC2939885

[33] Cronn R, Liston A, Parks M, Gernandt DS, Shen R, et al (2008) Multiplex sequencing of plant chloroplast genomes using Solexa sequencing-by-synthesis technology. Nucleic Acids Res 36: e122. 18753151 10.1093/nar/gkn502 PMC2577356

[34] Margulies M, Egholm M, Altman WE, Attiya S, Bader JS, et al (2005) Genome sequencing in microfabricated high-density picolitre reactors. Nature 437: 376–380. 16056220 10.1038/nature03959 PMC1464427

[35] Gordon D, Abajian C, Green P (1998) Consed: a graphical tool for sequence finishing. Genome Res 8: 195–202. 9521923 10.1101/gr.8.3.195

[36] Wyman SK, Jansen RK, Boore JL (2004) Automatic annotation of organellar genomes with DOGMA. Bioinformatics 20: 3252–3255. 15180927 10.1093/bioinformatics/bth352

[37] Altschul SF, Gish W, Miller W, Myers EW, Lipman DJ (1990) Basic local alignment search tool. J Mol Biol 215: 403–410. 2231712 10.1016/S0022-2836(05)80360-2

[38] Timme RE, Kuehl JV, Boore JL, Jansen RK (2007) A comparative analysis of the Lactuca and Helianthus (Asteraceae) plastid genomes: identification of divergent regions and categorization of shared repeats. Am J Bot 94: 302–312. 21636403 10.3732/ajb.94.3.302

[39] Dempewolf H, Kane NC, Ostevik KL, Geleta M, Barker MS, et al (2010) Establishing genomic tools and resources for Guizotia abyssinica (L.f.) Cass.-the development of a library of expressed sequence tags, microsatellite loci, and the sequencing of its chloroplast genome. Mol Ecol Resour 10: 1048–1058. 21565115 10.1111/j.1755-0998.2010.02859.x

[40] Kumar S, Hahn FM, McMahan CM, Cornish K, Whalen MC (2009) Comparative analysis of the complete sequence of the plastid genome of Parthenium argentatum and identification of DNA barcodes to differentiate Parthenium species and lines. BMC Plant Biol 9: 131. 19917140 10.1186/1471-2229-9-131 PMC2784773

[41] Nie X, Lv S, Zhang Y, Du X, Wang L, et al (2012) Complete chloroplast genome sequence of a major invasive species, crofton weed (Ageratina adenophora). PLoS One 7: e36869. 22606302 10.1371/journal.pone.0036869 PMC3350484

[42] Doorduin L, Gravendeel B, Lammers Y, Ariyurek Y, Chin AWT, et al (2011) The complete chloroplast genome of 17 individuals of pest species Jacobaea vulgaris: SNPs, microsatellites and barcoding markers for population and phylogenetic studies. DNA Res 18: 93–105. 21444340 10.1093/dnares/dsr002 PMC3077038

[43] Frazer KA, Pachter L, Poliakov A, Rubin EM, Dubchak I (2004) VISTA: computational tools for comparative genomics. Nucleic Acids Res 32: W273–279. 15215394 10.1093/nar/gkh458 PMC441596

[44] Cai Z, Penaflor C, Kuehl JV, Leebens-Mack J, Carlson JE, et al (2006) Complete plastid genome sequences of Drimys, Liriodendron, and Piper: implications for the phylogenetic relationships of magnoliids. BMC Evol Biol 6: 77. 17020608 10.1186/1471-2148-6-77 PMC1626487

[45] Hansen DR, Dastidar SG, Cai Z, Penaflor C, Kuehl JV, et al (2007) Phylogenetic and evolutionary implications of complete chloroplast genome sequences of four early-diverging angiosperms: Buxus (Buxaceae), Chloranthus (Chloranthaceae), Dioscorea (Dioscoreaceae), and Illicium (Schisandraceae). Mol Phylogenet Evol 45: 547–563. 17644003 10.1016/j.ympev.2007.06.004

[46] Kurtz S, Schleiermacher C (1999) REPuter: fast computation of maximal repeats in complete genomes. Bioinformatics 15: 426–427. 10366664 10.1093/bioinformatics/15.5.426

[47] Thiel T, Michalek W, Varshney RK, Graner A (2003) Exploiting EST databases for the development and characterization of gene-derived SSR-markers in barley (Hordeum vulgare L.). Theor Appl Genet 106: 411–422. 12589540 10.1007/s00122-002-1031-0

[48] Saski C, Lee SB, Fjellheim S, Guda C, Jansen RK, et al (2007) Complete chloroplast genome sequences of Hordeum vulgare, Sorghum bicolor and Agrostis stolonifera, and comparative analyses with other grass genomes. Theor Appl Genet 115: 571–590. 17534593 10.1007/s00122-007-0567-4 PMC2674615

[49] Goremykin VV, Hirsch-Ernst KI, Wolfl S, Hellwig FH (2003) Analysis of the Amborella trichopoda chloroplast genome sequence suggests that amborella is not a basal angiosperm. Mol Biol Evol 20: 1499–1505. 12832641 10.1093/molbev/msg159

[50] Jansen RK, Kaittanis C, Saski C, Lee SB, Tomkins J, et al (2006) Phylogenetic analyses of Vitis (Vitaceae) based on complete chloroplast genome sequences: effects of taxon sampling and phylogenetic methods on resolving relationships among rosids. BMC Evol Biol 6: 32. 16603088 10.1186/1471-2148-6-32 PMC1479384

[51] Tamura K, Peterson D, Peterson N, Stecher G, Nei M, et al (2011) MEGA5: molecular evolutionary genetics analysis using maximum likelihood, evolutionary distance, and maximum parsimony methods. Mol Biol Evol 28: 2731–2739. 21546353 10.1093/molbev/msr121 PMC3203626

[52] Young HA, Lanzatella CL, Sarath G, Tobias CM (2011) Chloroplast genome variation in upland and lowland switchgrass. PLoS One 6: e23980. 21887356 10.1371/journal.pone.0023980 PMC3161095

[53] Panero JL, Funk VA (2008) The value of sampling anomalous taxa in phylogenetic studies: major clades of the Asteraceae revealed. Mol Phylogenet Evol 47: 757–782. 18375151 10.1016/j.ympev.2008.02.011

[54] Edgar RC (2004) MUSCLE: a multiple sequence alignment method with reduced time and space complexity. BMC Bioinformatics 5: 113. 15318951 10.1186/1471-2105-5-113 PMC517706

[55] Wang W, Messing J (2011) High-throughput sequencing of three Lemnoideae (duckweeds) chloroplast genomes from total DNA. PLoS One 6: e24670. 21931804 10.1371/journal.pone.0024670 PMC3170387

[56] Nock CJ, Waters DL, Edwards MA, Bowen SG, Rice N, et al (2011) Chloroplast genome sequences from total DNA for plant identification. Plant Biotechnol J 9: 328–333. 20796245 10.1111/j.1467-7652.2010.00558.x

[57] Zhang T, Zhang X, Hu S, Yu J (2011) An efficient procedure for plant organellar genome assembly, based on whole genome data from the 454 GS FLX sequencing platform. Plant Methods 7: 38. 22126655 10.1186/1746-4811-7-38 PMC3248859

[58] Neckermann K, Zeltz P, Igloi GL, Kossel H, Maier RM (1994) The role of RNA editing in conservation of start codons in chloroplast genomes. Gene 146: 177–182. 8076816 10.1016/0378-1119(94)90290-9

[59] Kim KJ, Jansen RK (1995) ndhF sequence evolution and the major clades in the sunflower family. Proc Natl Acad Sci U S A 92: 10379–10383. 7479788 10.1073/pnas.92.22.10379 PMC40800

[60] Kim KJ, Choi KS, Jansen RK (2005) Two chloroplast DNA inversions originated simultaneously during the early evolution of the sunflower family (Asteraceae). Mol Biol Evol 22: 1783–1792. 15917497 10.1093/molbev/msi174

[61] Raubeson LA, Jansen RK (1992) Chloroplast DNA evidence on the ancient evolutionary split in vascular land plants. Science 255: 1697–1699. 17749424 10.1126/science.255.5052.1697

[62] Tangphatsornruang S, Uthaipaisanwong P, Sangsrakru D, Chanprasert J, Yoocha T, et al (2011) Characterization of the complete chloroplast genome of Hevea brasiliensis reveals genome rearrangement, RNA editing sites and phylogenetic relationships. Gene 475: 104–112. 21241787 10.1016/j.gene.2011.01.002

[63] Palmer JD, Nugent JM, Herbon LA (1987) Unusual structure of geranium chloroplast DNA: A triple-sized inverted repeat, extensive gene duplications, multiple inversions, and two repeat families. Proc Natl Acad Sci U S A 84: 769–773. 16593810 10.1073/pnas.84.3.769 PMC304297

[64] Ogihara Y, Terachi T, Sasakuma T (1988) Intramolecular recombination of chloroplast genome mediated by short direct-repeat sequences in wheat species. Proc Natl Acad Sci U S A 85: 8573–8577. 3186748 10.1073/pnas.85.22.8573 PMC282501

[65] Hiratsuka J, Shimada H, Whittier R, Ishibashi T, Sakamoto M, et al (1989) The complete sequence of the rice (Oryza sativa) chloroplast genome: intermolecular recombination between distinct tRNA genes accounts for a major plastid DNA inversion during the evolution of the cereals. Mol Gen Genet 217: 185–194. 2770692 10.1007/BF02464880

[66] Provan J, Powell W, Hollingsworth PM (2001) Chloroplast microsatellites: new tools for studies in plant ecology and evolution. Trends Ecol Evol 16: 142–147. 11179578 10.1016/s0169-5347(00)02097-8

[67] Jakobsson M, Sall T, Lind-Hallden C, Hallden C (2007) Evolution of chloroplast mononucleotide microsatellites in Arabidopsis thaliana. Theor Appl Genet 114: 223–235. 17123063 10.1007/s00122-006-0425-9

[68] Ebert D, Peakall R (2009) Chloroplast simple sequence repeats (cpSSRs): technical resources and recommendations for expanding cpSSR discovery and applications to a wide array of plant species. Mol Ecol Resour 9: 673–690. 21564725 10.1111/j.1755-0998.2008.02319.x

[69] Provan J, Corbett G, Waugh R, McNicol JW, Morgante M, et al (1996) DNA fingerprints of rice (Oryza sativa) obtained from hypervariable chloroplast simple sequence repeats. Proc Biol Sci 263: 1275–1281. 8914327 10.1098/rspb.1996.0187

[70] Provan J, Russell JR, Booth A, Powell W (1999) Polymorphic chloroplast simple sequence repeat primers for systematic and population studies in the genus Hordeum. Mol Ecol 8: 505–511. 10199011 10.1046/j.1365-294x.1999.00545.x

[71] Provan J, Corbett G, McNicol JW, Powell W (1997) Chloroplast DNA variability in wild and cultivated rice (Oryza spp.) revealed by polymorphic chloroplast simple sequence repeats. Genome 40: 104–110. 9061917 10.1139/g97-014

[72] Bryan GJ, McNicol JW, Meyer RC, Ramsay G, De Jong WS (1999) Polymorphic simple sequence repeat markers in chloroplast genomes of Solanaceous plants. Theoretical and Applied Genetics 99: 859–867.

[73] Flannery ML, Mitchell FJ, Coyne S, Kavanagh TA, Burke JI, et al (2006) Plastid genome characterisation in Brassica and Brassicaceae using a new set of nine SSRs. Theor Appl Genet 113: 1221–1231. 16909279 10.1007/s00122-006-0377-0

[74] Provan J (2000) Novel chloroplast microsatellites reveal cytoplasmic variation in Arabidopsis thaliana. Mol Ecol 9: 2183–2185. 11123644

[75] Denda T, Watanabe K, Kosuge K, Yahara T, Ito M (1999) Molecular phylogeny of Brachycome (Asteraceae) Plant Systematics And Evolution. 217: 299–311.

[76] Fernandez IA, Aguilar JF, Panero JL, Feliner GN (2001) A phylogenetic analysis of Doronicum (Asteraceae, Senecioneae) based on morphological, nuclear ribosomal (ITS), and chloroplast (trnL-F) evidence. Mol Phylogenet Evol 20: 41–64. 11421647 10.1006/mpev.2001.0954

[77] Terry R, Brown G, Olmstead R (1997) Examination of subfamilial phylogeny in Bromeliaceae using comparative sequencing of the plastid locus ndhF. Am J Bot 84: 664. 21708619

[78] Scotland RW, Sweere JA, Reeves PA, Olmstead RG (1995) Higher-level systematics of Acanthaceae determined by Chloroplast DNA sequences American Journal of Botany. 82: 266–275.

[79] Catalan P, Kellogg EA, Olmstead RG (1997) Phylogeny of Poaceae subfamily Pooideae based on chloroplast ndhF gene sequences. Mol Phylogenet Evol 8: 150–166. 9299221 10.1006/mpev.1997.0416

[80] Palmer JD, Jansen RK, Michaels HJ, Chase MW, Manhart JR (1988) Chloroplast DNA Variation and Plant Phylogeny. Annals of the Missouri Botanical Garden 75: 1180–1206.

[81] Kajita T, Kamiya K, Nakamura K, Tachida H, Wickneswari R, et al (1998) Molecular phylogeny of Dipetrocarpaceae in Southeast Asia based on nucleotide sequences of matK, trnL intron, and trnL-trnF intergenic spacer region in chloroplast DNA. Mol Phylogenet Evol 10: 202–209. 9878231 10.1006/mpev.1998.0516

[82] Lee JH, Lee JW, Sung JS, Bang KH, Moon SG (2009) Molecular authentication of 21 Korean artemisia species (Compositae) by polymerase chain reaction-restriction fragment length polymorphism based on trnL-F region of chloroplast DNA. Biol Pharm Bull 32: 1912–1916. 19881307 10.1248/bpb.32.1912

[83] Tamura K, Nei M (1993) Estimation of the number of nucleotide substitutions in the control region of mitochondrial DNA in humans and chimpanzees. Mol Biol Evol 10: 512–526. 8336541 10.1093/oxfordjournals.molbev.a040023

[84] Daniell H, Lee SB, Grevich J, Saski C, Quesada-Vargas T, et al (2006) Complete chloroplast genome sequences of Solanum bulbocastanum, Solanum lycopersicum and comparative analyses with other Solanaceae genomes. Theor Appl Genet 112: 1503–1518. 16575560 10.1007/s00122-006-0254-x

[85] Schulze-Menz GK (1964) A. Engler’s: Syllabus der Pflanzenfamilien. Berlin: Gebrüder Borntraeger.

