# Supplementary figures and images for: Complete Chloroplast Genome Sequences of Mongolia Medicine Artemisia frigida and Phylogenetic Relationships with Other Plants
Source: PLoS One. 2013 Feb 27;8(2):e57533. doi: 10.1371/journal.pone.0057533 (PMC3583863; doi:10.1371/journal.pone.0057533)

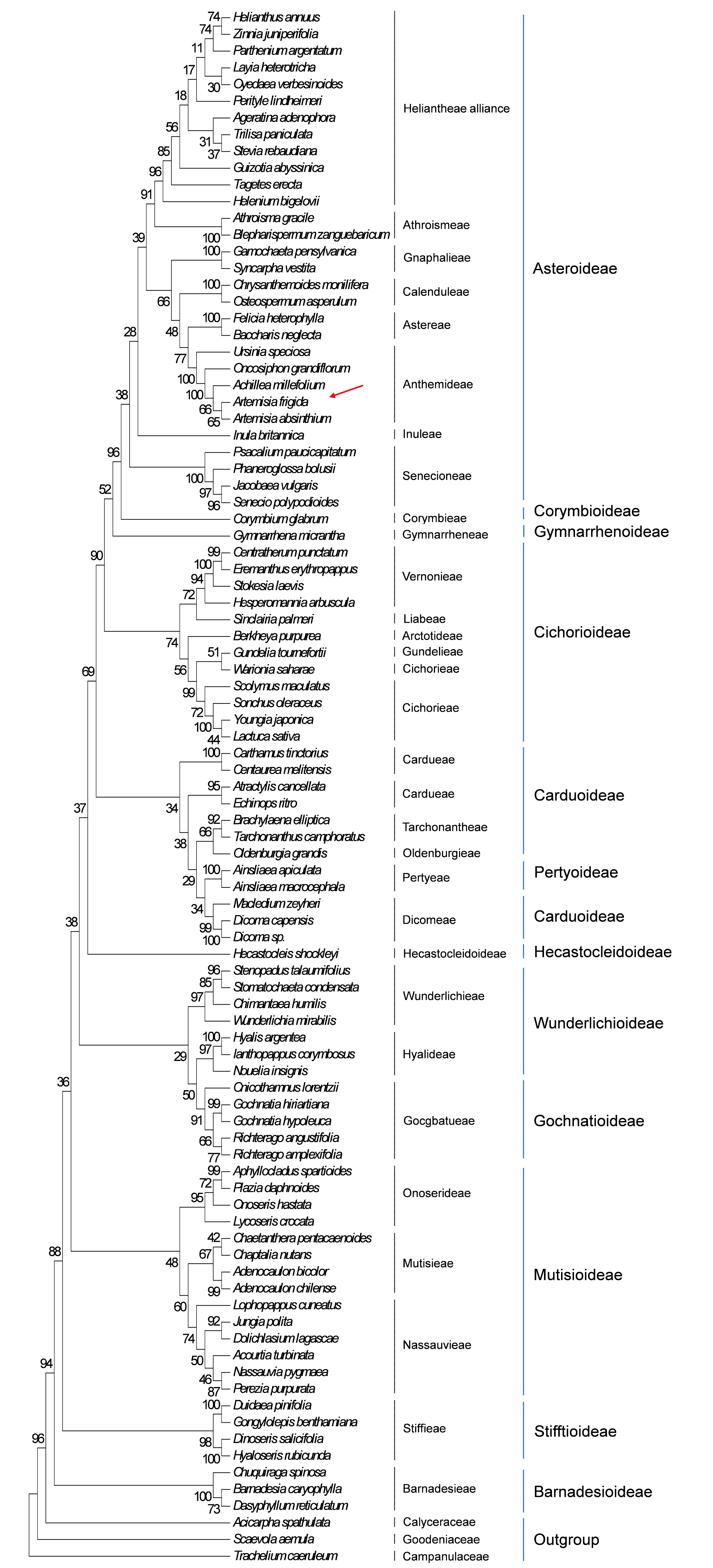

Supplement: Figure S1 — Reconstruction of phylogentic tree of Asteraceae and related families. The tree topology was constructed with the maximum likelihood method using the ndhF and trnL-F gene sequence regions. Bootstrp proportions shown above the branches. lnL = −20049.26. The position of the sequenced Artemisia frigida species is indicated with a red arrow. (TIF) [file pone.0057533.s001.tif]
