# Supplementary material for: Complete Chloroplast Genome Sequences of Mongolia Medicine Artemisia frigida and Phylogenetic Relationships with Other Plants
Source: PLoS One. 2013 Feb 27;8(2):e57533. doi: 10.1371/journal.pone.0057533 (PMC3583863; doi:10.1371/journal.pone.0057533)
Supplement: Table S1 — List of primer pairs used in sequence verification and improvement of the Artemisia frigida chloroplast genome. (DOC) [file pone.0057533.s002.doc]

**Table S1 A whole list of all pairs of primers used in *Artemisia frigida* chloroplast**

| **Primers ID** | **sequence** | **Primers ID** | **sequence** |
| --- | --- | --- | --- |
| ^a^1F | ATCGCTTTTTGCATGTCCTC | 1R | CTAAGCGTTGGCTCGGTAAG |
| 2F | CGAATATGGAATTCAAAGGG | 2R | AGTGGATTGGATCGACTTGG |
| 3F | CCGTCCCGAAGAGTAACTAGG | 3R | AGGTATCGGGAAGGAATTGG |
| 4F | TTAGCATGGCGTACTCCTCC | 4R | TACTGCGCTCTCCAAGTGTG |
| 5F | CTGCGGAAAAATAGCTCGAC | 5R | AAAGATGCAGTCATCGATTCTC |
| ^b^6F | CAAGTTCAATGTTAGCGAGATTAG | 6R | TTTTTCAGTTAGTATAGCCTCTTTCG |
| 7F | ACTGCATCTTTACAAAATTCATACC | 7R | TTGTGGTATTCCACCTCTTGC |
| 8F | GGTTAATCCCGCGGTAGAC | 8R | GAAGGGCCGACTCCTATTTC |
| 9F | TGGGGTATGACTATGAAAAGAGG | 9R | TTTGAACAATTTTGAAAAGACTCAG |
| 10F | GCTTTTTGACAAGCCTTTGC |  |  |
| 11F | TGGGATTTATTGTTTTCCATTTC | 11R | GAAAGACATCTATTGTTCAAAACG |
| 12F | ATTCTATCGTTGCAAGAGTCG | 12R | GCCCATACTGCTCCTGAAAG |
| 13F | CATAACTCGTTCCTGCCAAG | 13R | TGATTTCCGTTGGAAATTAGG |
| 14F | AACCCGCAAATATTGGTAAAACTAC | 14R | CGTAGGAGCCATAAATGTTTTAATC |
| 15F | CCTCATACGGCTCCTCAAAG | 15R | GAGTACTTCCCACGATTCCG |
| 16F | CCATGAATTGCTGAAAGCG | 16R | TAATTATGATTTGCTTGTTCCTG |
| 17F | TTGCTTCTTTATTGTTTCTAATGATGA | 17R | AAAGGATTCGATAATCAGCACTC |
| 18F | GCTGATTATCGAATCCTTTTCTG | 18R | CACTCAAAGGCGTAAAATGG |
| 19F | ATCCAGCCCTTGCATTATG | 19R | CGACAAAATGGAAGAAGAAGAAG |
| ^c^20F | CCTTGGGGTTATCCTGCAC | 20R | AACCGTGCTAACCTTGGTATG |
| 21F | GCGTCTCTCTAAAATTGCAGTC | 21R | GAAGGCGGATTTGGTATTTG |
| 22F | AATCCTTACTACATCAACATTTCAGA | 22R | ATAAAAGATGCTTCCTCTTTGC |
| 23F | CAATCAGAGGCATAATTGGAAC |  |  |
| 24F | ATCCCAATTATGACACTCATGG | 24R | AGGGCTAGATAGATCTCAACAACAC |
| 25F | TAAGAACGGAAGCTCGTTGC | 25R | GGCTCGGTACAAAGCCTTC |
| 26F | AAATAAAACCTATGGGACGG | 26R | TGATTCAAATGATGGGTTCG |
| 27F | ACGGCTCGAGAAAAATGATG | 27R | CGCAGCTAAATAAAAATGACTATGAG |
|  |  | 28R | GGTTGGGAAGTTGAAAATGG |
| 29F | GATCTTGGAGGTTGTGTAATGC | 29R | GGCCTGGCTAGGTATTGACC |
| 30F | CGGCCGAGAATTCCACTAC | 30R | TGTTATTCATGATATTGATCCGATTC |
| 31F | GCGAAGTGGAAAAAGACAGG | 31R | GGGATTTTCTATCAAGTGATTCG |
| 32F | TTCTGTTTCGGTTGCATCTC | 32R | ATCGTCAAGCGTACCCATTC |
| 33F | CTGTACCTGACATGTTTCTTGG | 33R | ATTCCCTGTGGGTTCTTCTTC |
| 34F | GCCCTTCACTTTCATTACATCG | 34R | TTCGATGGGAGTTTCTCTTG |
| 35F | TGACATGTACTGGTCGTTGG | 35R | GGTGGTATCAATCGGAATGC |
| 36F | CATCAAATGAACCTAACAACATTCC | 36R | TACCATTTTAGGATTCCATTTCC |
| 37F | TGGTATTCAAGCTCTTATTTTTGC | 37R | ACCTTGACCAACTCCAGGTC |
| 38F | ATCGAACAGCTGGAAAAAGC | 38R | TGAATACGAGCAATGCCATC |
| 39F | CCAAGACATTCACCGAGGAC | 39R | CGCTACCCGCTCTATACCCT |
| 40F | TTCGTTCTCTATTTCTATTTGAGAGG | 40R | GGAATTTTTAGTGATTCAACAGATG |
| 41F | AAAGGAATTGATCGTGATTTTG | 41R | CCGGAGACTCTCTTCAGATTG |
| 42F | GCAACAAACATGCATGAACC | 42R | GCAAGGTTGGTTGGATTAGC |
| 43F | AACGGACCTTTTGGATCTTC | 43R | GACCAAGCCGCTGAGTATTG |
| 44F | TTCCCTTCTGCTGCCTATTG | 44R | GGTGGACTATTCCAGTAAGATGG |
| 45F | TTGGCTATTCATATTCGCTATTC | 45R | AGATTTGAACTGGTGACACGAG |
| 46F | GGGACGGGATCATAATGAGA |  |  |
| 47F | ACATGTCAATACTGGCAACAATG |  |  |
| 48F | TTCGCTTCTTTTTGATTTCTCC | 48R | TGGATAGGATGGCCTTTACG |
| 49F | AAGCAGAAACATAGATGCACTCC | 49R | CCTCCATCGAGATTGGATTC |
| 50F | GCACTCCCCTGAAAAACATC | 50R | GGTGCGTCCGAATAATCATC |
| 51F | GCTACATCTAGTACCGGACC | 51R | AACCCCAGCTTTGAATCC |
| 52F | CTTTCTACCCATCCTGTATATTGTC | 52R | ATGATCATTGTCAATCTCAAAAATATG |
| 53F | ATGCATGTGGCTATCTTTAGTG | 53R | TGAATCTTATTCGTCGACTTTATG |
| 54F | GGCCAGAACTTCTTGTTTCAG | 54R | ACACCCCTTTTCTTGTGTCG |
| 55F | TTGGAGTGAACCTATTGCGG | 55R | ACACGTAAGTTCGCGGAAAG |
| 56F | AAACTAACCAAACCATGGATAAATC | 56R | TGAGTTGGAAGGAATCCACC |
| 57F | TAGACCCCGATCGGTAAATG | 57R | TGACATCGTGATTTGGATTG |
| 58F | CCCAATTTTGATTTGGACATATAG | 58R | GTTCATAGGAACAAAGAGAAGCAG |
| 59F | CCCAGATTTGGATGCTCAAG | 59R | GAAACAGCAACCCTAGTCGC |
| 60F | CAATTGTGAAATAAAATATCATGACG | 60R | CGAGCTATTCGCAGAAGTGG |
| 61F | TCGGCATTTCGTATAGAGGT | 61R | AATAGCAGCGTCCAAAATGC |
| 62F | CAACGCCAGAGGATGTTTTC | 62R | AAATTCGTGGGCGTTCCTAC |
| ^d^63F | CTAAAAATGGGTGAAGGTATTCTACC | 63R | TGCGCCTCTGCATCTAGC |
| 64F | AAAAACATCCATGCCCAGAC | 64R | TCAATGCGGGTCGAAGTATC |
| 65F | ACGAGTAGTGGGGTCCTGAG | 65R | CAGTCAGATGTTTGGGGCAG |
| A.f.P1F | TCATGAAATGGATTGGAAATATATA | A.f.P1R | CAAAATGGTTAACCCCGTCT |
| A.f.P2F | TTTCCATCACTTTGGCATGT | A.f.P2R | AATGGATTGGATTGCTATTAGTTC |
| H.a.P1F | GATTGCTATTAGTTTGGATACGG | H.a.P1R | TTTCCATCACTTTGGCATGT |
| H.a.P2F | TTTCAGTCAGTATAGCCTGTTTCG | H.a.P2R | TGGATTGCTATTAAAGAATTAAAGAC |
| L.s.P1F | CGAGATTAGTCAACATTTTTATGTTTC | L.s.P1R | AATGGACGATTCCATCGATTA |
| L.s.P2F | TCCTTTCAATCAAGACGTTG | L.s.P2R | AGATTAGTCAACAAGATTCAAACATTG |

A.f.: primers for *Artemisia frigida*, H.a.:primers for *Helianthus annuus*, L.s.:primers for *Lactuca sativa*

^a^primers used to verify junction of LSC/IRb.

^b^primers used to verify junction of IRb/SSC.

^c^primers used to verify junction of SSC/IRa.

^d^primers used to verify junction of IRa/LSC.
