# Supplementary material for: Complete Chloroplast Genome Sequences of Mongolia Medicine Artemisia frigida and Phylogenetic Relationships with Other Plants
Source: PLoS One. 2013 Feb 27;8(2):e57533. doi: 10.1371/journal.pone.0057533 (PMC3583863; doi:10.1371/journal.pone.0057533)
Supplement: Table S2 — The codon-anticondon recognition pattern and codon usage for Artemisia frigida chloroplast genome. (DOC) [file pone.0057533.s003.doc]

| **Table S2 The codon-anticodon recognition pattern and codon usage for *Artemisia frigida* chloroplast genome** | | | | | | | | | |
| --- | --- | --- | --- | --- | --- | --- | --- | --- | --- |
| **Animo acid** | **Codon** | **No.** | **RSCU** | **tRNA** | **Animo acid** | **Codon** | **No.** | **RSCU** | **tRNA** |
| Phe | UUU | 979 | 1.32 |  | Tyr | UAU | 800 | 1.63 |  |
| Phe | UUC | 504 | 0.68 | trnF-GAA | Tyr | UAC | 180 | 0.37 | trnY-GUA |
| Leu | UUA | 880 | 1.87 | trnL-UAA | Ter | UAA | 49 | 1.69 |  |
| Leu | UUG | 578 | 1.23 | trnL-CAA | Ter | UAG | 23 | 0.79 |  |
| Leu | CUU | 621 | 1.32 |  | Ter | UGA | 15 | 0.52 |  |
| Leu | CUC | 188 | 0.4 |  | His | CAU | 467 | 1.52 |  |
| Leu | CUA | 366 | 0.78 | trnL-UAG | His | CAC | 146 | 0.48 | trnH-GUG |
| Leu | CUG | 191 | 0.41 |  | Gln | CAA | 732 | 1.52 | trnQ-UUG |
| Ile | AUU | 1078 | 1.46 |  | Gln | CAG | 233 | 0.48 |  |
| Ile | AUC | 430 | 0.58 | trnI-GAU | Asn | AAU | 1008 | 1.56 |  |
| Ile | AUA | 700 | 0.95 | trnI CAU | Asn | AAC | 285 | 0.44 | trnN-GUU |
| Met | AUG | 625 | 1 | trnM-CAU | Lys | AAA | 1026 | 1.47 | trnK-UUU |
| Val | GUU | 506 | 1.44 |  | Lys | AAG | 366 | 0.53 |  |
| Val | GUC | 172 | 0.49 | trnV-GAC | Asp | GAU | 860 | 1.6 |  |
| Val | GUA | 538 | 1.53 | trnV-UAC | Asp | GAC | 213 | 0.4 | trnD-GUC |
| Val | GUG | 187 | 0.53 |  | Glu | GAA | 994 | 1.49 | trnE-UUC |
| Ser | UCU | 584 | 1.74 |  | Glu | GAG | 343 | 0.51 |  |
| Ser | UCC | 320 | 0.95 | trnS-GGA | Ser | AGU | 415 | 1.24 |  |
| Ser | UCA | 412 | 1.23 | trnS-UGA | Ser | AGC | 118 | 0.35 | trnS-GCU |
| Ser | UCG | 164 | 0.49 |  | Cys | UGU | 201 | 1.4 | trnC-GCA |
| Pro | CCU | 442 | 1.59 |  | Cys | UGC | 87 | 0.6 |  |
| Pro | CCC | 188 | 0.67 | trnP-UGG | Trp | UGG | 459 | 1 | trnW-CCA |
| Pro | CCA | 324 | 1.16 |  | Arg | CGU | 350 | 1.34 | trnR-ACG |
| Pro | CCG | 161 | 0.58 |  | Arg | CGC | 105 | 0.4 |  |
| Thr | ACU | 533 | 1.63 |  | Arg | CGA | 340 | 1.3 |  |
| Thr | ACC | 241 | 0.74 | trnT-GGU | Arg | CGG | 121 | 0.46 |  |
| Thr | ACA | 410 | 1.25 | trnT-UGU | Arg | AGA | 478 | 1.83 | trnR UCU |
| Thr | ACG | 127 | 0.39 |  | Arg | AGG | 174 | 0.67 |  |
| Ala | GCU | 615 | 1.74 |  | Gly | GGU | 584 | 1.31 |  |
| Ala | GCC | 227 | 0.64 |  | Gly | GGC | 188 | 0.42 | trnG-GCC |
| Ala | GCA | 412 | 1.17 | trnA-UGC | Gly | GGA | 701 | 1.58 |  |
| Ala | GCG | 158 | 0.45 |  | Gly | GGG | 304 | 0.68 |  |
