# Supplementary material for: Complete Chloroplast Genome Sequences of Mongolia Medicine Artemisia frigida and Phylogenetic Relationships with Other Plants
Source: PLoS One. 2013 Feb 27;8(2):e57533. doi: 10.1371/journal.pone.0057533 (PMC3583863; doi:10.1371/journal.pone.0057533)
Supplement: Table S3 — Size comparison of Artemisia frigida chloroplast genomic regions with those in other species of Asteraceae. (DOC) [file pone.0057533.s004.doc]

**Table S3 Size comparison of *Artemisia frigida* chloroplast genomic**

regions with those in other species of Asteraceae.

|  | **Length (bp)** | | | |
| --- | --- | --- | --- | --- |
| **Plant species** | **Total genome** | **LSC** | **SSC** | **IR** |
| *Jacobaea vulgaris* | 150686 | 82855 | 18277 | 24777 |
| *Ageratina adenophora* | 150698 | 84829 | 18359 | 23755 |
| *Artemisia frigida* | 151076 | 82740 | 18394 | 24971 |
| [*Helianthus annuus*](http://en.wikipedia.org/wiki/Ageratina) | 151104 | 83530 | 18308 | 24633 |
| *Guizotia abyssinica* | 151762 | 83636 | 18228 | 24950 |
| *Latuca sativa* | 152772 | 84105 | 18599 | 25034 |
| *Parthenium argentatum* | 152803 | 84566 | 19390 | 24424 |

LSC, Large Single Copy; SSC, Small Single Copy; IR, Inverted Repeat
