# Supplementary material for: Complete Chloroplast Genome Sequences of Mongolia Medicine Artemisia frigida and Phylogenetic Relationships with Other Plants
Source: PLoS One. 2013 Feb 27;8(2):e57533. doi: 10.1371/journal.pone.0057533 (PMC3583863; doi:10.1371/journal.pone.0057533)
Supplement: Table S4 — The GenBank accession numbers of all the 58 cp genomes used for phylogenetic analysis. (DOC) [file pone.0057533.s005.doc]

| **Table S4 The GenBank accession numbers of all the 58 cp genomes used for phylogenetic analysis** | | |
| --- | --- | --- |
| **No.** | **Taxon** | **GenBank Accession No.** |
|  | Gymnosperm Outgroup |  |
| 1 | *Pinus thunbergii* | NC_001631 |
| 2 | *Ginkgo biloba* | NC_016986 |
|  | Basal Angiosperms |  |
| 3 | *Chloranthus spicatus* | NC_009598 |
| 4 | *Illicium oligandrum* | NC_009600 |
| 5 | *Nymphaea alba* | NC_006050 |
| 6 | *Nuphar advena* | NC_008788 |
|  | Magnoliids |  |
| 7 | *Magnolia kwangsiensis* | NC_015892 |
| 8 | *Drimys granadensis* | NC_008456 |
| 9 | *Calycanthus floridus* | NC_004993 |
|  | Monocots |  |
| 10 | *Acorus americanus* | NC_010093 |
| 11 | *Colocasia esculenta* | NC_016753 |
| 12 | *Dioscorea elephantipes* | NC_009601 |
| 13 | *Elaeis guineensis* | NC_017602 |
| 14 | *Typha latifolia* | NC_013823 |
| 15 | *Zea mays* | NC_001666 |
| 16 | *Sorghum bicolor* | NC_008602 |
| 17 | *Triticum aestivum* | NC_002762 |
| 18 | *Hordeum vulgare* | NC_008590 |
|  | Eudicots |  |
| 19 | *Buxus microphylla* | NC_009599 |
| 20 | *Platanus occidentalis* | NC_008335 |
| 21 | *Nelumbo nucifera* | NC_015610 |
| 22 | *Nandina domestica* | NC_008336 |
| 23 | *Ranunculus macranthus* | NC_008796 |
|  | Asterids |  |
| 24 | *Ageratina adenophora* | NC_015621 |
| 25 | *Guizotia abyssinica* | NC_010601 |
| 26 | *Helianthus annuus* | NC_007977 |
| 27 | *Lactuca sativa* | NC_007578 |
| 28 | *Panax ginseng* | NC_006290 |
| 29 | *Eleutherococcus senticosu* | NC_016430 |
| 30 | *Oxypolis greenmanii* | NC_015832 |
| 31 | *Crithmum maritimum* | NC_015804 |
| 32 | *Petroselinum crispum* | NC_015821 |
| 33 | *Sesamum indicum* | NC_016433 |
| 34 | *Jasminum nudiflorum* | NC_008407 |
| 35 | *Coffea arabica* | NC_008535 |
| 36 | *Ipomoea purpurea* | NC_009808 |
| 37 | *Nicotiana tabacum* | NC_001879 |
| 38 | *Atropa belladpmma* | NC_004561 |
| 39 | *Solanum bulbocastanum* | NC_007943 |
| 40 | *Solanum lycopersicum* | NC_007898 |
|  | *Caryophyllales* |  |
| 41 | *Fagopyrum esculentum* | NC_010776 |
| 42 | *Spinacia oleracea* | NC_002202 |
| 43 | *Silene latifolia* | NC_016730 |
|  | Rosids |  |
| 44 | *Vitis vinifera* | NC_007957 |
| 45 | *Arabidopsis thaliana* | NC_000932 |
| 46 | *Brassica napus* | NC_016734 |
| 47 | *Gossypium raimondii* | NC_016668 |
| 48 | *Citrus sinensis* | NC_008334 |
| 49 | *Erodium carvifolium* | NC_015083 |
| 50 | *Hevea brasiliensis* | NC_015308 |
| 51 | *Manihot esculenta* | NC_010433 |
| 52 | *Ricinus communis* | NC_016736 |
| 53 | *Millettia pinnata* | NC_016708 |
| 54 | *Prunus persica* | NC_014697 |
| 55 | *Fragaria vesca* | NC_015206 |
| 56 | *Castanea mollissima* | NC_014674 |
| 57 | *Cucumis sativus* | NC_007144 |
| 58 | *Corynocarpus laevigata* | NC_014807 |
