# Supplementary material for: Complete Chloroplast Genome Sequences of Mongolia Medicine Artemisia frigida and Phylogenetic Relationships with Other Plants
Source: PLoS One. 2013 Feb 27;8(2):e57533. doi: 10.1371/journal.pone.0057533 (PMC3583863; doi:10.1371/journal.pone.0057533)
Supplement: Table S5 — The GenBank accession numbers of ndhF gene and trnL-F gene region from all the 92 species used for phylogenetic analysis. (DOC) [file pone.0057533.s006.doc]

| **Table S5 The GenBank accession numbers of *ndhF* gene and *trnL-F* gene region from all the 92 species used for phylogenetic analysis** | | | | | |
| --- | --- | --- | --- | --- | --- |
| **No.** | **Subfamily name** | **Tribe name** | **Species name** | ***ndhF* gene accession No** | ***trnL-F* accession No.** |
| 1 | Asteroideae | Anthemideae | *Artemisia absinthium* | EU334460 | FJ710526 |
| 2 |  | Anthemideae | *Achillea millefolium* | EU385124 | AY603266 |
| 3 |  | Anthemideae | *Oncosiphon grandiflorum* | EU385189 | EU385095 |
| 4 |  | Anthemideae | *Ursinia speciosa* | EU385215 | EU385121 |
| 5 |  | Astereae | *Felicia heterophylla* | EU385161 | EU385068 |
| 6 |  | Astereae | *Baccharis neglecta* | EU384949 | EU385041 |
| 7 |  | Athroismeae | *Athroisma gracile* | L39455 | AY216019/AY216144 |
| 8 |  | Athroismeae | *Blepharispermum zanguebaricum* | L39456 | AY216022/AY216147 |
| 9 |  | Calenduleae | *Chrysanthemoides monilifera* | EU385145 | EU385052 |
| 10 |  | Calenduleae | *Osteospermum asperulum* | EU385191 | EU385097 |
| 11 |  | Gnaphalieae | *Gamochaeta pensylvanica* | EU385162 | EU385070 |
| 12 |  | Gnaphalieae | *Syncarpha vestita* | EU385211 | EU385117 |
| 13 |  | Heliantheae alliance | *Stevia rebaudiana* | AF384787 | AY216117/AY216242 |
| 14 |  | Heliantheae alliance | *Trilisa paniculata* | AF384744 | AY216198 |
| 15 |  | Heliantheae alliance | *Ageratina adenophora* | extract from NC_015621 | extract from NC_015621 |
| 16 |  | Heliantheae alliance | *Helenium bigelovii* | AF384730 | AY216057/AY216182 |
| 17 |  | Heliantheae alliance | *Helianthus annuus* | extract from NC_007977 | extract from NC_007977 |
| 18 |  | Heliantheae alliance | *Oyedaea verbesinoides* | AF384758 | AY216088/AY216213 |
| 19 |  | Heliantheae alliance | *Parthenium argentatum* | extract from NC_013553 | extract from NC_013553 |
| 20 |  | Heliantheae alliance | *Zinnia juniperifolia* | AF384805 | AY216135/AY216260 |
| 21 |  | Heliantheae alliance | *Layia heterotricha* | AF384742 | AY216071/AY216196 |
| 22 |  | Heliantheae alliance | *Guizotia abyssinica* | extract from NC_010601 | extract from NC_010601 |
| 23 |  | Heliantheae alliance | *Perityle lindheimeri* | AF384761 | AY216092/AY216217 |
| 24 |  | Heliantheae alliance | *Tagetes erecta* | L39466 | AY216119/AY216244 |
| 25 |  | Inuleae | *Inula britannica* | AF384737 | AY216190 |
| 26 |  | Senecioneae | *Senecio polypodioides* | EU385205 | EU385111 |
| 27 |  | Senecioneae | *Jacobaea vulgaris* | extract from NC_015543 | extract from NC_015543 |
| 28 |  | Senecioneae | *Phaneroglossa bolusii* | AF384765 | EF538116 |
| 29 |  | Senecioneae | *Psacalium paucicapitatum* | EU385200 | EU385106 |
| 30 | Barnadesioideae | Barnadesieae | *Barnadesia caryophylla* | L39394 | AY504768 |
| 31 |  | Barnadesieae | *Chuquiraga spinosa* | EU385146 | EU385053 |
| 32 |  | Barnadesieae | *Dasyphyllum reticulatum* | EU385150 | EU385057 |
| 33 | Carduoideae | Cardueae | *Atractylis cancellata* | EU385134 | EU385040 |
| 34 |  | Cardueae | *Carthamus tinctorius* | EU385139 | EU385046 |
| 35 |  | Cardueae | *Centaurea melitensis* | EU385140 | EU385047 |
| 36 |  | Cardueae | *Echinops ritro* | EU385158 | EU385065 |
| 37 |  | Dicomeae | *Dicoma capensis* | EU385152 | EU385059 |
| 38 |  | Dicomeae | *Dicoma sp.* | EU385151 | EU385058 |
| 39 |  | Dicomeae | *Macledium zeyheri* | EU385184 | EU385090 |
| 40 |  | Oldenburgieae | *Oldenburgia grandis* | EU385188 | EU385094 |
| 41 |  | Tarchonantheae | *Brachylaena elliptica* | EU385138 | EU385045 |
| 42 |  | Tarchonantheae | *Tarchonanthus camphoratus* | EU385212 | EU385118 |
| 43 | Cichorioideae | Arctotideae | *Berkheya purpurea* | EU385136 | EU385043 |
| 44 |  | Cichorieae | *Youngia japonica* | EU385218 | EU385123 |
| 45 |  | Cichorieae | *Sonchus oleraceus* | EU385206 | EU385112 |
| 46 |  | Cichorieae | *Lactuca sativa* | extract from DQ383816 | extract from DQ383816 |
| 47 |  | Cichorieae | *Scolymus maculatus* | EU385204 | EU385110 |
| 48 |  | Cichorieae | *Warionia saharae* | EU385216 | AY702089/AY702090 |
| 49 |  | Gundelieae | *Gundelia tournefortii* | EU385169 | EU385075 |
| 50 |  | Liabeae | *Sinclairia palmeri* | EU385181 | EU385087 |
| 51 |  | Vernonieae | *Centratherum punctatum* | EU385141 | EU385048 |
| 52 |  | Vernonieae | *Hesperomannia arbuscula* | EU385172 | EU385078 |
| 53 |  | Vernonieae | *Eremanthus erythropappus* | EU385159 | EU385066 |
| 54 |  | Vernonieae | *Stokesia laevis* | EU385209 | EU385115 |
| 55 | Corymbioideae | Corymbieae | *Corymbium glabrum* | EU385148 | EU385055 |
| 56 | Gochnatioideae | Gochnatieae | *Cnicothamnus lorentzii* | EU385147 | EU385054 |
| 57 |  | Gochnatieae | *Gochnatia hiriartiana* | EU385166 | EU385072 |
| 58 |  | Gochnatieae | *Gochnatia hypoleuca* | EU385165 | EU385071 |
| 59 |  | Gochnatieae | *Richterago amplexifolia* | EU385202 | EU385108 |
| 60 |  | Gochnatieae | *Richterago angustifolia* | EU385127 | EU385033 |
| 61 | Gymnarrhenoideae | Gymnarrheneae | *Gymnarrhena micrantha* | EU385170 | EU385076 |
| 62 | Hecastocleidoideae | Hecastocleidoideae | *Hecastocleis shockleyi* | EU385171 | EU385077 |
| 63 | Mutisioideae | Mutisieae | *Adenocaulon bicolor* | EU385129 | EU385035 |
| 64 |  | Mutisieae | *Adenocaulon chilense* | EU385128 | EU385034 |
| 65 |  | Mutisieae | *Chaetanthera pentacaenoides* | EU385142 | EU385049 |
| 66 |  | Mutisieae | *Chaptalia nutans* | EU385143 | EU385050 |
| 67 |  | Nassauvieae | *Acourtia turbinata* | EU385126 | EU385032 |
| 68 |  | Nassauvieae | *Dolichlasium lagascae* | EU385155 | EU385062 |
| 69 |  | Nassauvieae | *Jungia polita* | EU385178 | EU385084 |
| 70 |  | Nassauvieae | *Lophopappus cuneatus* | EU385182 | EU385088 |
| 71 |  | Nassauvieae | *Nassauvia pygmaea* | EU385186 | EU385092 |
| 72 |  | Nassauvieae | *Perezia purpurata* | EU385194 | EU385100 |
| 73 |  | Onoserideae | *Aphyllocladus spartioides* | EU385132 | EU385038 |
| 74 |  | Onoserideae | *Lycoseris crocata* | EU385183 | EU385089 |
| 75 |  | Onoserideae | *Onoseris hastata* | EU385190 | EU385096 |
| 76 |  | Onoserideae | *Plazia daphnoides* | EU385197 | EU385103 |
| 77 | Pertyoideae | Pertyeae | *Ainsliaea apiculata* | EU385130 | EU385036 |
| 78 |  | Pertyeae | *Ainsliaea macrocephala* | EU385131 | EU385037 |
| 79 | Stifftioideae | Stifftieae | *Dinoseris salicifolia* | EU385154 | EU385061 |
| 80 |  | Stifftieae | *Duidaea pinifolia* | EU385157 | EU385064 |
| 81 |  | Stifftieae | *Gongylolepis benthamiana* | EU385167 | EU385073 |
| 82 |  | Stifftieae | *Hyaloseris rubicunda* | EU385176 | EU385082 |
| 83 | Wunderlichioideae | Hyalideae | *Hyalis argentea* | EU385175 | EU385081 |
| 84 |  | Hyalideae | *Ianthopappus corymbosus* | EU385177 | EU385083 |
| 85 |  | Hyalideae | *Nouelia insignis* | EU385187 | EU385093 |
| 86 |  | Wunderlichieae | *Chimantaea humilis* | EU385144 | EU385051 |
| 87 |  | Wunderlichieae | *Stenopadus talaumifolius* | EU385207 | EU385113 |
| 88 |  | Wunderlichieae | *Stomatochaeta condensata* | EU385210 | EU385116 |
| 89 |  | Wunderlichieae | *Wunderlichia mirabilis* | EU385217 | EU385122 |
|  | **Outgroup** |  |  |  |  |
| 90 | Calyceraceae |  | *Acicarpha spathulata* | EU385125 | EU385031 |
| 91 | Campanulaceae |  | *Trachelium caeruleum* | extract from NC_010442 | extract from NC_010442 |
| 92 | Goodeniaceae |  | *Scaevola aemula* | EU385203 | EU385109 |
